# Tongqiao Huoxue Decoction ameliorates traumatic brain injury‐induced gastrointestinal dysfunction by regulating CD36/15‐LO/NR4A1 signaling, which fails when CD36 and CX3CR1 are deficient

**DOI:** 10.1111/cns.14247

**Published:** 2023-05-08

**Authors:** Chunzhu Wei, Feng Zhu, Jintao Yu, Fei Gao, Yuyi Yuan, Yanlong Zhang, Xinjie Liu, Si Chu, Dandan Cui, Heng Fan, Wenzhu Wang

**Affiliations:** ^1^ Department of Integrated Traditional and Western Medicine, Union Hospital, Tongji Medical College Huazhong University of Science and Technology Wuhan China; ^2^ Department of Otolaryngology, Union Hospital, Tongji Medical College Huazhong University of Science and Technology Wuhan China

**Keywords:** CD36/15‐LO/NR4A1 signaling, gastrointestinal dysfunction, intestinal mucosal barrier, Tongqiao Huoxue Decoction, traumatic brain injury

## Abstract

**Aims:**

Gastrointestinal (GI) dysfunction, as a common peripheral‐organ complication after traumatic brain injury (TBI), is primarily characterized by gut inflammation and damage to the intestinal mucosal barrier (IMB). Previous studies have confirmed that TongQiao HuoXue Decoction (TQHXD) has strong anti‐inflammatory properties and protects against gut injury. However, few have reported on the therapeutic effects of TQHXD in a TBI‐induced GI dysfunction model. We aimed to explore the effects of TQHXD on TBI‐induced GI dysfunction and the underlying mechanism thereof.

**Methods:**

We assessed the protective effects and possible mechanism of TQHXD in treating TBI‐induced GI dysfunction via gene engineering, histological staining, immunofluorescence (IF), 16S ribosomal ribonucleic acid (rRNA) sequencing, real‐time polymerase chain reaction (RT‐PCR), enzyme‐linked immunosorbent assay (ELISA), Western blot (WB), and flow cytometry (FCM).

**Results:**

TQHXD administration ameliorated TBI‐induced GI dysfunction by modulating the abundance and structure of bacteria; reconstructing the destroyed epithelial and chemical barriers of the IMB; and improving M1/M2 macrophage, T‐regulatory cell (Treg)/T helper 1 cell (Th_1_), as well as Th_17_/Treg ratios to preserve homeostasis of the intestinal immune barrier. Notably, Cluster of Differentiation 36 (CD36)/15‐lipoxygenase (15‐LO)/nuclear receptor subfamily 4 group A member 1 (NR4A1) signaling was markedly stimulated in colonic tissue of TQHXD‐treated mice. However, insufficiency of both CD36 and (C‐X3‐C motif) chemokine receptor 1 (CX3CR1) worsened GI dysfunction induced by TBI, which could not be rescued by TQHXD.

**Conclusion:**

TQHXD exerted therapeutic effects on TBI‐induced GI dysfunction by regulating the intestinal biological, chemical, epithelial, and immune barriers of the IMB, and this effect resulted from the stimulation of CD36/NR4A1/15‐LO signaling; however, it could not do so when CX3CR1 and CD36 were deficient. TQHXD might therefore be a potential drug candidate for treating TBI‐induced GI dysfunction.

## Graphical abstract

TBI led to gastrointestinal dysfunction, which further caused the damaged intestinal biological, chemical, epithelial, and immune barriers. TQHXD could recover the damaged intestinal biological, chemical, epithelial, and immune barriers via the stimulation of CD36/NR4A1/15‐LO signal in mice with TBI‐induced gastrointestinal dysfunction.

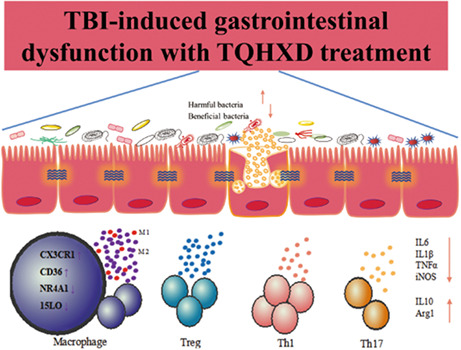

## 1 INTRODUCTION

Traumatic brain injury (TBI) is a complicated condition well known to be not only injurious to the brain but also detrimental to numerous organ systems, including the gastrointestinal (GI),^1^ cardiovascular,^2^ and respiratory systems.^3^ Increasing evidence suggests that TBI can result in GI dysfunction, which primarily refers to intestinal inflammation and destruction of the intestinal mucosal barrier (IMB).^4^, ^5^, ^6^ Furthermore, GI dysfunction can cause a lack of appetite,^7^ increased rates of bacterial translocation and infection,^8^ and disorders of the immune system,^9^ which ultimately raise the mortality rate of TBI patients.^10^ Currently, the pathogenesis of TBI‐induced GI dysfunction is unclear, and no effective therapy is available. Therefore, it is extremely urgent to look for optimal drugs.

The IMB is composed of a biological barrier, an epithelial barrier, a chemical barrier, and an immune barrier, which collectively protect the gut wall from attack by bacteria and other harmful materials.^11^ One of the most important properties is disordered microbiota, which can cause imbalance of intestinal immune moderation, affecting the repair of intestinal epithelial cells (ECs) and associated mucus secretion, and leading to the worsening of intestinal inflammation.^12^

The anomalous activation of immunity response is characteristic of the pathological process after TBI.^13^ In the intestine, macrophages, T helper 1 and 17 cells (Th_1_, Th_17_), and T‐regulatory cells (Tregs) play crucial roles in modulating innate immune and adaptive responses as well as inflammatory reactions. Macrophage subsets, but not dendritic cells (DCs), in the murine intestinal lamina propria (LP) have been found to preferentially express (C‐X3‐C motif) chemokine receptor 1 (CX3CR1). CX3CR1 deficiency increases the susceptibility of mice to dextran sulfate sodium (DSS)‐induced gut inflammation by reducing the count of macrophages in the LP, unbalancing gut dysbacteriosis, upregulating T cells that are positive for interferon‐gamma (IFN‐γ^+^)/interleukin‐17A (IL17A^+^)/cluster of differentiation 4 (CD4^+^), and downregulating forkhead box protein 3–positive (FoxP3^+^)/CD4^+^ T cells.^14^ However, modulation of such T cells in a model of CX3CR1‐deficient mice with TBI‐induced GI dysfunction has not been reported.

The central role of CX3CR1^+^ macrophages in the intestine has been illustrated by direct depletion of CX3CR1 from macrophages,^15^ which can modulate intestinal homeostasis by mediating co‐action between gut microbiota and host immunity in the healthy as well as the inflammatory state.^16^ Furthermore, our previous study confirmed that CX3CR1 deficiency aggravated the white matter injury and affected CD36/15‐lipoxygenase (15‐LO)/nuclear receptor subfamily 4 group A member 1 (NR4A1) signaling in mice with TBI.^17^ Therefore, we here studied TBI‐induced GI dysfunction and the status of CD36/15‐LO/NR4A1 signaling in CX3CR1‐deficient mice by observing the biological, immune, epithelial, and chemical barriers of the IMB.

TongQiao HuoXue Decoction (TQHXD), a classic prescription for treating brain disease that dates from China's Qing Dynasty, originates from an important textbook, named Yi Lin Gai Cuo.^18^ Moreover, TQHXD is a designated prescription for the treatment of brain diseases in the diagnosis and treatment guide of common diseases in traditional Chinese medicine^19^ and has strong effects against inflammation^20^ and oxidative stress (OS) injury.^21^ There are many modern studies on TQHXD, mainly focusing on the areas of TBI,^20^, ^22^ stroke,^23^, ^24^ and cerebral ischemia/reperfusion injury (IRI).^25^, ^26^ For instance, Zhang et al.^27^ found that TQHXD could maintain intestinal homeostasis by regulating the balance of Bacteroidetes, *Allobaculum*, *Bifidobacterium*, and *Lactobacillus*; reducing IL‐17 levels; and increasing IL‐10 levels in a stroke model. However, there are no studies on TQHXD's ameliorative effect on TBI‐induced intestinal inflammation, and the mechanism thereof needs to be further clarified.

**FIGURE 1:**
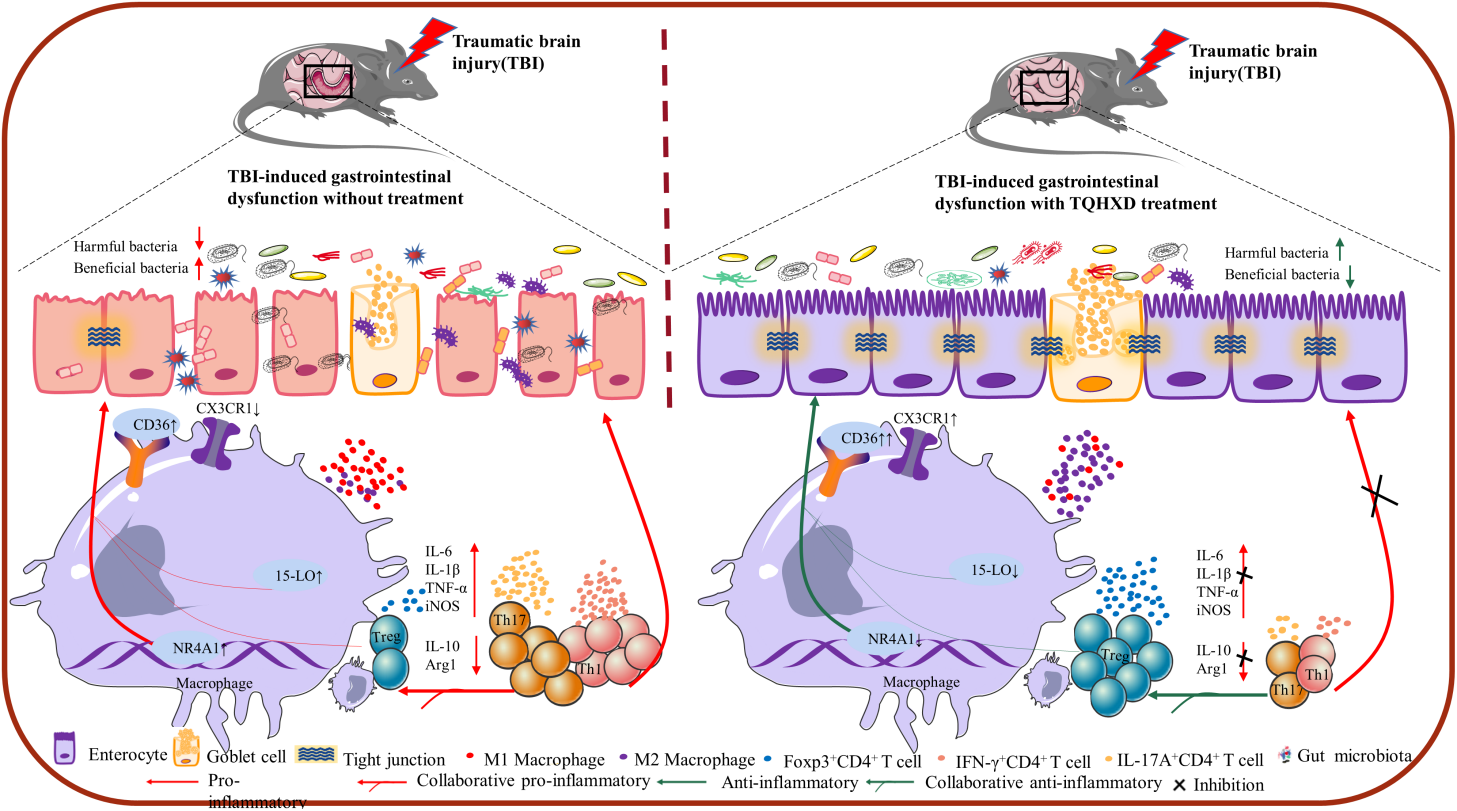
Schematic depiction about the therapy effects of TQHXD by regulating CD36/15LO/NR4A1 signaling pathway in mice with TBI‐induced gastrointestinal dysfunction.

In this study, we investigated the therapeutic effect of TQHXD on TBI‐induced intestinal inflammation and destruction of the IMB. In addition, we used CX3CR1 knockout (KO) mice and the CD36 inhibitor sulfo‐N‐succinimidyl oleate (SSO) to systematically analyze and validate the mechanism of CD36/15‐LO/NR4A1 signaling from the perspectives of the biological, chemical, epithelial, and immune barriers of the IMB in TBI mice (Figure 1).

## 2 MATERIALS AND METHODS

### 2.1 Animals

Dr. Jonathan Bromburg originally generated 8‐ to 10‐week‐old male CX3CR1^green fluorescent protein(GFP)/GFP^ and age‐matched CX3CR1^GFP/+^ C57BL/6 mice (University of Maryland, Baltimore, MD, USA). Tail snips of these mice were subjected to genotyping at the CX3CR1 gene via polymerase chain reaction (PCR) amplification. Eight‐week‐old C57BL/6 male mice were purchased from Beijing Vital River Laboratory. We housed all mice in specific pathogen‐free (SPF) conditions at the Animal Center of Tongji Medical College (TMC), Huazhong University of Science and Technology. All animal experiments were approved by the Institutional Animal Care and Use Committee of TMC. Throughout the experiment, we made all efforts to minimize the animals' mental and physical discomfort.

### 2.2 Experimental model of traumatic brain injury

Based on Feeney's free‐fall TBI model,^28^ we anesthetized mice with 4% isoflurane in oxygen. Briefly, after anesthesia, the head was fixed onto a stereotactic frame, the skull was exposed, and the scalp was incised. Along the sagittal suture, 1 mm caudal to coronal and 1 mm left of the sagittal suture, we trephined a craniotomy (5 mm in diameter) into the skull over the left parietal cortex using a portable drill. A 40‐g mass was dropped along a plastic injury cannula and struck the 3‐cm‐diameter cylinder placed atop the craniotomy at a velocity of 5.5 m/s to a depth of 5 mm. We closed the scalp with bone wax before suturing. Mice in the Sham group received a scalp incision, but the skull was left intact. Throughout the modeling process, mice were kept on a temperature‐controlled heating bed that maintained their body temperature at 37°C.

### 2.3 Preparation of TQHXD aqueous extract and drug delivery

We purchased TQHXD from Hubei Chenmei Chinese Medicine Co. Ltd. TQHXD is composed of the dry contents of five herbal medicines (*Carthamus tinctorius* L., *Prunus persica* (L.) *Batsch*, *Ligusticum chuanxiong Hort*., *Paeonia veitchii Lynch*, *Moschus berezovskii Flerov*) at a 9:6:6:6:0.15 mass ratio. Batch numbers were 2,020,009,101, 2,020,010,101, 2,020,912,105, 2,020,006,103, and 20,200,418, respectively. After immersion in distilled water for 30 min, TQHXD was first boiled for 30 min at 1:10 w/v. We then collected the once‐boiled liquid, added fresh distilled water, and boiled the decoction again for 30 min at 1:8 w/v. Then, *Moschus berezovskii Flerov* T was added to the twice‐boiled decoction, followed by filtering and concentrating to 1 g/mL and lyophilization to produce lyophilized TQHXD powder.

Mice were administered TQHXD at a gavaged dose of 3 g/kg/day according to the human‐to‐mouse body surface area conversion formula after recovery from anesthesia. We administered the CD36 inhibitor SSO (Cat. No. HY‐112847; MedChemExpress [MCE], Shanghai, China), daily per the manufacturer's instructions. Sham group mice were gavaged with an equal volume of distilled water.

### 2.4 Identification of active compounds in TQHXD by HPLC–MS/MS

We detected phytochemicals of TQHXD via high‐performance liquid chromatography with tandem mass spectrometry (HPLC–MS/MS). In brief, the supernatant of TQHXD was collected after settling at 4°C for 24 h. We added 200 mg supernatant to a 1‐mL cocktail of water and methanol (2:8, v/v) and then centrifuged the mixture at 12,000 ***g*** for 20 min at 4°C. The suspension was subjected to HPLC–MS/MS analysis after being filtered through a 0.22‐μm strainer. In addition, we precisely weighed and dissolved six standard substances (kaempferol, CAS No. 520–18‐3; paeoniflorin, CAS No. 23180‐57‐6; amygdalin, CAS No. 29883–15‐6; ferulic acid, CAS No. 1135‐24‐6; hydroxysafflor yellow A, CAS No. 78281–02‐4; all from MCE; as well as musk ketone, CAS No. 200418, Xizang) in methanol to provide a blended standard solution of 10 μg/mL. Phytochemical analysis of TQHXD was performed on an UltiMate 3000 RS system (Thermo Fisher Scientific), and compounds were separated using a high‐resolution mass spectrometer (Thermo Fisher).

### 2.5 Assessment of TBI‐induced inflammation and intestinal mucosal barrier injury and sample collection

We recorded body weight (BW), stool character, and stool blood daily to assess the severity of inflammation. On day 3 after TBI, mice were anesthetized and perfused transcardially with iced phosphate‐buffered saline (PBS) followed by 4% paraformaldehyde. Colonic tissue at a distance of 1.5 cm from the anus was isolated, rinsed, soaked in 4% paraformaldehyde for 24 h, and placed in 30% sucrose for 72 h at 4°C. Then, we embedded the tissue in an optimal cutting temperature compound for pathological experiments. The remaining colon was excised, cut lengthwise, washed with pre‐cooled PBS, and then stored at −80°C for further research.

### 2.6 Histopathological evaluation

We took coronal sections (20 mm) at 100‐mm intervals. Sections were mounted onto slides and subjected to hematoxylin and eosin (H&E) and periodic acid–Schiff (PAS) staining to assess histological score^29^ and level of mucus secretion, respectively.

### 2.7 Fecal‐DNA extraction and microbial identification

Three days before and after TBI, we collected 100 mg fecal samples from each mouse in sterile 1.5‐ml tubes and stored them at −80°C. An E.Z.N.A. Stool deoxyribonucleic acid (DNA) Kit (Cat. No. D4015; Omega Bio‐tek, Inc.) was used to purify fecal DNA. After fecal‐DNA extraction, we identified counts of total fecal bacteria and specific bacteria via PCR. The relative primer sequences are listed in Table 1. Mice were sacrificed immediately for the collection of colon tissue to detect PCR, WB, inflammatory mediators, etc.

**TABLE 1:** Primer sequences for RT‐PCR.

Gene		Primer sequences (5′‐3′)
16S rRNA	Sense	CCTACGGRRBGCASCAGKVRVGAAT
	Anti‐sense	GGACTACNVGGGTWTCTAATCC
β‐Actin	Sense	GTGACGTTGACATCCGTAAAGA
	Anti‐sense	GTAACAGTCCGCCTAGAAGCAC
ZO‐1	Sense	GTTGGTACGGTGCCCTGAAAGA
	Anti‐sense	GCTGACAGGTAGGACAGACGAT
Occludin	Sense	TGGCAAGCGATCATACCCAGAG
	Anti‐sense	CTGCCTGAAGTCATCCACACTC
CD36	Sense	GCCAAGCTATTGCGACATGA
	Anti‐sense	TCTGGATTCTGGAGGGGTGA
15‐LO	Sense	GAGATCACTGAGATTGGGTTGC
	Anti‐sense	CCTTGGTTTTAGGTGGTGG
NR4A1	Sense	GCTTCGTGTCAGCACTATGGG
	Anti‐sense	GCAGATGTACTTGGCGCTTTT
iNOS	Sense	GAGACAGGGAAGTCTGAAGCAC
	Anti‐sense	CCAGCAGTAGTTGCTCCTCTTC
Arg1	Sense	CATTGGCTTGCGAGACGTAGAC
	Anti‐sense	GCTGAAGGTCTCTTCCATCACC

### 2.8 Real‐time polymerase chain reaction

We extracted total ribonucleic acid (RNA) from colonic tissues using TRIzol reagent (Cat. No. G3013; Servicebio Technology). After RNA isolation, PrimeScript RT Master Mix (Cat. No. RR036A; TaKaRa Bio) was applied to generate complementary DNA (cDNA). Next, we used SYBR Premix ExTaq (Cat. No. RR036A; TaKaRa) to amplify target cDNA fragments and confirm gene expression levels. The housekeeping gene β‐actin was used to normalize the expression of target genes in colonic tissue. We calculated the results using 2^−△△Ct^. Relative primer sequences are listed in Table 1.

### 2.9 Cytokine detection

To determine concentrations of tumor necrosis factor alpha (TNF‐α), IL‐6, IL‐1β, and IL‐10 in mouse colons, we used ELISA kits (Cat Nos. 012022, 122,021, 112,021, and 102,021, respectively; Yanzun Biotechnology) per manufacturer's instructions. Briefly, colonic tissue was homogenized in grinders; after centrifugation at 3000 *g* for 15 min, we subjected colonic tissue supernatants to ELISA to detect concentrations of the above cytokines.

### 2.10 Western blot

To extract protein from every 40 mg of colonic tissue, 400 μL of radioimmunoprecipitation assay (RIPA) lysis buffer with a strong cracking effect (Cat. No. G2002; Servicebio) and 1% protease inhibitor cocktail (Cat. No. G2006; Servicebio;) were required. We collected the lysate into another tube after homogenization and centrifugation and then detected concentrations in the supernatant using a Bicinchoninic Acid (BCA) Kit (Cat. No. P1010; Beyotime Institute of Biotechnology). The loading buffer was added into the supernatant after ensuring that the concentrations of each sample were the same, and the mixture was then heated at 95°C for 10 min. We separated total proteins via sodium dodecyl sulfate polyacrylamide gel electrophoresis (SDS–PAGE) and then transferred them onto 0.45‐ or 0.22‐μm polyvinylidene fluoride (PVDF) membranes (Cat. No. R1SB97913; MilliporeSigma), which were blocked with 8% skim milk at room temperature (RT) for 1 h and then incubated in primary antibodies (aBs) at 4°C for 8 h. The membranes were washed with tris‐buffered saline + Polysorbate 20 (TBST) buffer and incubated in secondary aBs at RT for 1 h. Finally, we visualized the membranes using a chemiluminescence system (iBright 750; Invitrogen) and analyzed them using ImageJ 2× software (National Institutes of Health [NIH]). Primary aBs and their dilution ratios were as follows: β‐actin, 1:1000 (Cat. No. GB15003; Servicebio); CD36, 1:1000 (Cat. No. A19016; ABclonal Technology); 15‐LO, 1:800 (Cat. No. NB120‐11197; Santa Cruz Biotechnology); NR4A1, 1:1000 (Cat. No. 25851‐1‐AP; Proteintech); inducible nitric oxide synthase (iNOS), 1:1000 (Cat. No. A3200; ABclonal); and arginase‐1 (Arg1), 1:1000 (Cat. No. A1847; ABclonal).

### 2.11 Immunofluorescence staining

Immunofluorescence (IF) staining was performed as described previously.^29^ After blocking the colon sections, we incubated them with rabbit anti‐ZO‐1 (1:300, Cat. No. 21773‐1‐AP; Proteintech) or rabbit anti‐occludin (1:200, Cat. No. 66378‐1‐Ig; Proteintech) at 4°C for 10 h and then with fluorescence‐labeled secondary aBs (1:100, Cat. Nos. GB21403 and GB22403; Servicebio) for 1 h at RT. A laser confocal scanning microscope (Olympus FV1000; Olympus) was used to observe expression levels of ZO‐1 and occludin.

### 2.12 Isolation of cells from lamina propria, spleen, and mesenteric lymph node; and flow cytometry

The colon was removed longitudinally and cleaned with Roswell Park Memorial Institute (RPMI) 1640 buffer containing 5% serum to wash out the contents of the intestinal lumen. We then cut the colon into the smallest pieces possible and placed them in an Eppendorf (EP; Eppendorf) tube containing digestive enzymes (2 mg/mL collagenase IV; Thermo Fisher; Cat. No. 17104019; and 0.25 mg/mL DNaseI; Roche; Cat. No. 10104159001). Then, tissues were transferred to a 15‐mL centrifuge tube, digestive enzymes were added to a volume of 5 mL, and the mixture was incubated in a water bath at 37°C with agitation for 30 min. Next, we used RPMI 1640 buffer containing 5% serum to end the digestion reaction and filtered the cell suspensions through a 100‐μm strainer. After filtration, the suspensions were centrifugated at 500 ***g*** for 10 min. Finally, we used a buffer of pre‐cooled PBS containing 2% bovine serum albumin (BSA) to re‐suspend the LP cells. Spleen and mesenteric lymph node (MLN) cells were isolated as previously described.^30^ Briefly, we placed spleen and MLN tissues in 1.5‐mL tubes with pre‐cooled PBS containing 2% BSA, which were then grounded, filtered into 50‐mL centrifuge tubes, and centrifugated at 500 ***g*** for 5 min. Next, the supernatants were removed, and erythrocyte lysate was added to the tubes to dissociate erythrocytes. Finally, we removed the supernatants again and used a pre‐cooled PBS buffer containing 2% BSA to re‐suspend the cells.

We used an anti‐CD45 antibody to identify lymphocytes, an anti‐CD4 antibody to label T‐cell subsets, and anti‐CD11b and anti‐F4/80 aBs to identify macrophages. Live cells were labeled using a Fixable Viability Stain Kit (Cat. No. 423101; BioLegend CNS, Inc.). All surface markers were incubated in the dark at 4°C for 30 min. Next, we fixed the surface‐stained cells and permeated them using a nuclear penetration fluid (Cat. No. 87‐8724‐00; Thermo Fisher) per the manufacturer's protocol. Anti‐CD45, anti‐CD11b, anti‐F4/80, anti‐CD11c, and anti‐CD206 aBs were stained for macrophages, and anti–IL‐17A, IFN‐γ, and *Foxp3* aBs were stained for T‐cell subsets, for 1.5 h at 4°C. After washing them, we re‐suspended the cells in PBS and analyzed them using CytExpert software (Beckman Coulter Life Sciences).

### 2.13 Statistical analysis

We used SPSS version 21.0 (IBM Corp.) and GraphPad Prism 8 software (GraphPad Software, Inc.) to perform statistical analysis. The Shapiro–Wilk test was used to test whether quantitative data accorded with a normal distribution and analysis of variance (ANOVA) was used to compare quantitative data among more than two groups, followed by a least significant difference (LSD) test for post hoc comparisons. We used an independent *t*‐test to compare quantitative data between the two groups. Non‐normally distributed data were expressed as medians and evaluated using the Mann–Whitney *U* test. *p* < 0.05 was considered statistically significant.

## 3 RESULTS

### 3.1 Major components of TQHXD as indicated by HPLC–MS/MS

**FIGURE 2:**
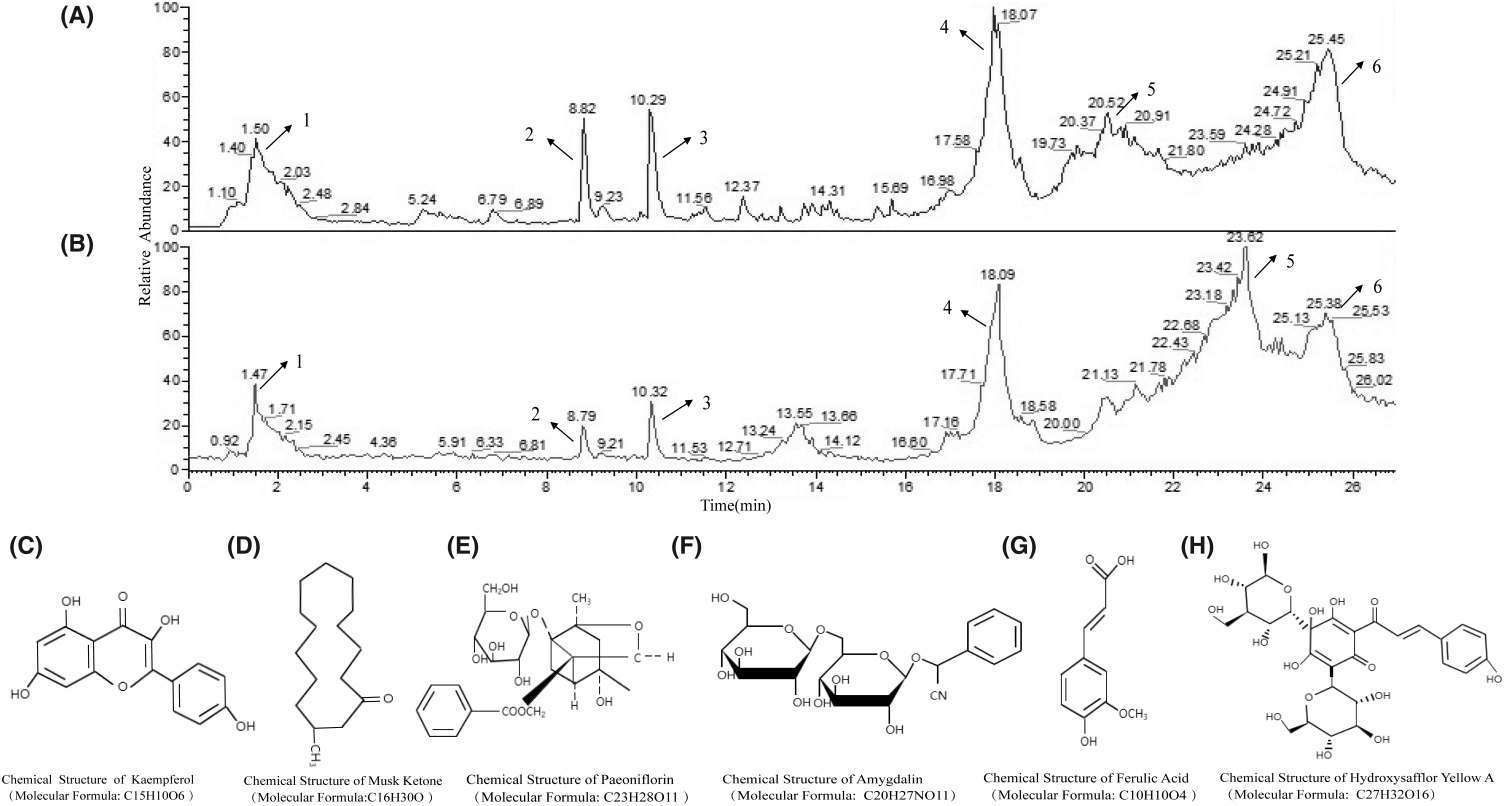
Major components of TQHXD as indicated by HPLC–MS/MS. (A) TQHXD chromatography. Peaks 1, 2, 3, 4, 5, and 6 were Kaempferol, Musk Ketone, Paeoniflorin, Amygdalin, Ferulic Acid, Hydroxysafflor Yellow A, respectively. (B) Chromatographic peaks came from the standards. 1, 2, 3, 4, 5, and 6 were Kaempferol, Musk Ketone, Paeoniflorin, Amygdalin, Ferulic Acid, Hydroxysafflor Yellow A, respectively. The molecular structure of Kaempferol (C), Musk Ketone (D), Paeoniflorin (E), Amygdalin (F), Ferulic Acid (G), Hydroxysafflor Yellow A (H) were described.

We identified the six constituents of TQHXD by comparing retention time and peak areas with those of the standards. A representative diagram of the six active ingredients in the five herbs is shown in Figure 2A–H. HPLC–MS/MS analysis of TQHXD revealed six typical peaks, numbered 1–6, which were kaempferol, musk ketone, paeoniflorin, amygdalin, ferulic acid, and hydroxysafflor yellow A.

### 3.2 TQHXD ameliorated TBI‐induced intestinal inflammation in mice

**FIGURE 3:**
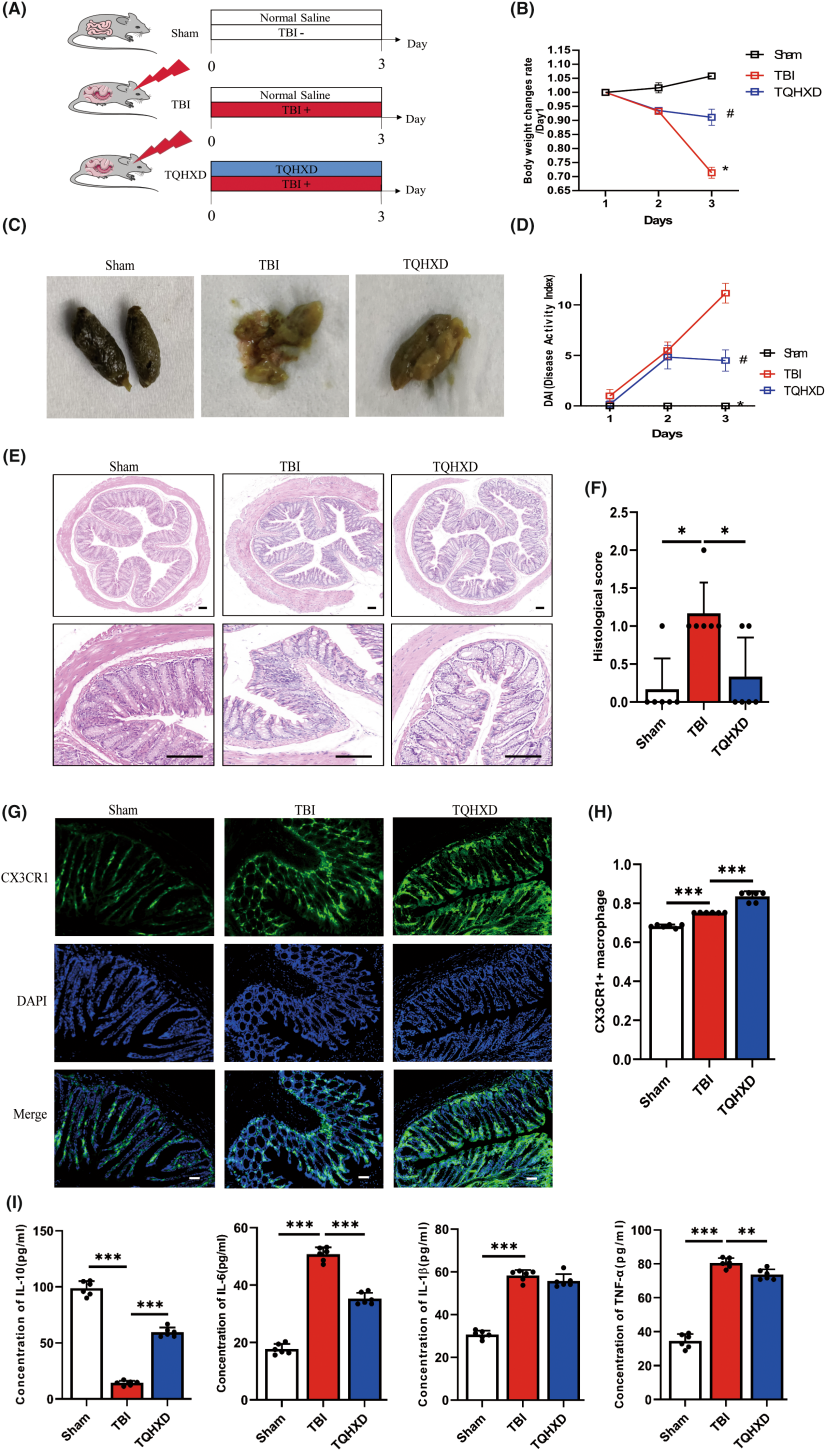
TQHXD ameliorated TBI‐induced intestinal inflammation in mice. (A) Schematic diagram illustrates the experimental design. (B) Changes in body weight in each group. (C) Representative photograph of feces. (D) DAI score. (E) HE staining of colon tissue (magnification above ×40, magnification below ×200). (F) Histological scores of colon tissue of each group. (G, H) The expression of green fluorescent protein of CX3CR1 on macrophages (magnification ×200). (I) The levels of cytokines including IL‐10, IL‐6, IL‐1β, and TNF‐α. Data were presented as mean ± SD. *p < 0.05, **p < 0.01, ***p < 0.001, significantly different as indicated.

To investigate the therapeutic effect of TQHXD on TBI‐induced intestinal inflammation and gut barrier impairment, we used the free‐fall method to construct a TBI‐induced GI dysfunction model (Figure 3A). The BW of mice in the TBI group decreased dramatically on Day 3 of the experiment (Figure 3B). In contrast, BW significantly improved in mice treated with TQHXD. Similarly, compared with the Sham group, the clinical symptoms of diarrhea and/or blood and disease activity index (DAI) score, which indicated the severity of intestinal inflammation, visibly increased in the TBI group, whereas such changes were markedly lower in the TQHXD group (Figure 3C,D). In addition, H&E staining detected fewer goblet cells, disordered glandular structure, and infiltration of inflammatory cells in the TBI group, which was in sharp contrast with results in the Sham group. Nevertheless, TQHXD therapy could rescue those changes and reduce histopathological scores (Figure 3E,F). To measure CX3CR1 expression in macrophages, we used CX3CR1 reporter mice, in which CX3CR1 is replaced with a green fluorescent protein (GFP) on macrophages in one allele without disrupting CX3CR1's functions, to further verify the degree of inflammation in colon tissue after TBI. Our results showed that compared with the Sham group, CX3CR1^+^ cell count increased in the TBI group and showed a further increase in the TQHXD group (Figure 3G,H). In line with the above results, compared with the Sham group, concentrations of pro‐inflammatory cytokines IL‐6, IL‐1β, and TNF‐α were clearly elevated in the TBI group, while the level of anti‐inflammatory cytokine IL‐10 showed the opposite tendency, and TQHXD could significantly reverse these changes (Figure 3I). Taken together, these data illustrated that TQHXD exerted therapeutic effects on TBI‐induced intestinal inflammation.

### 3.3 TQHXD relieved TBI‐induced inflammation in a microbiota‐dependent manner

**FIGURE 4:**
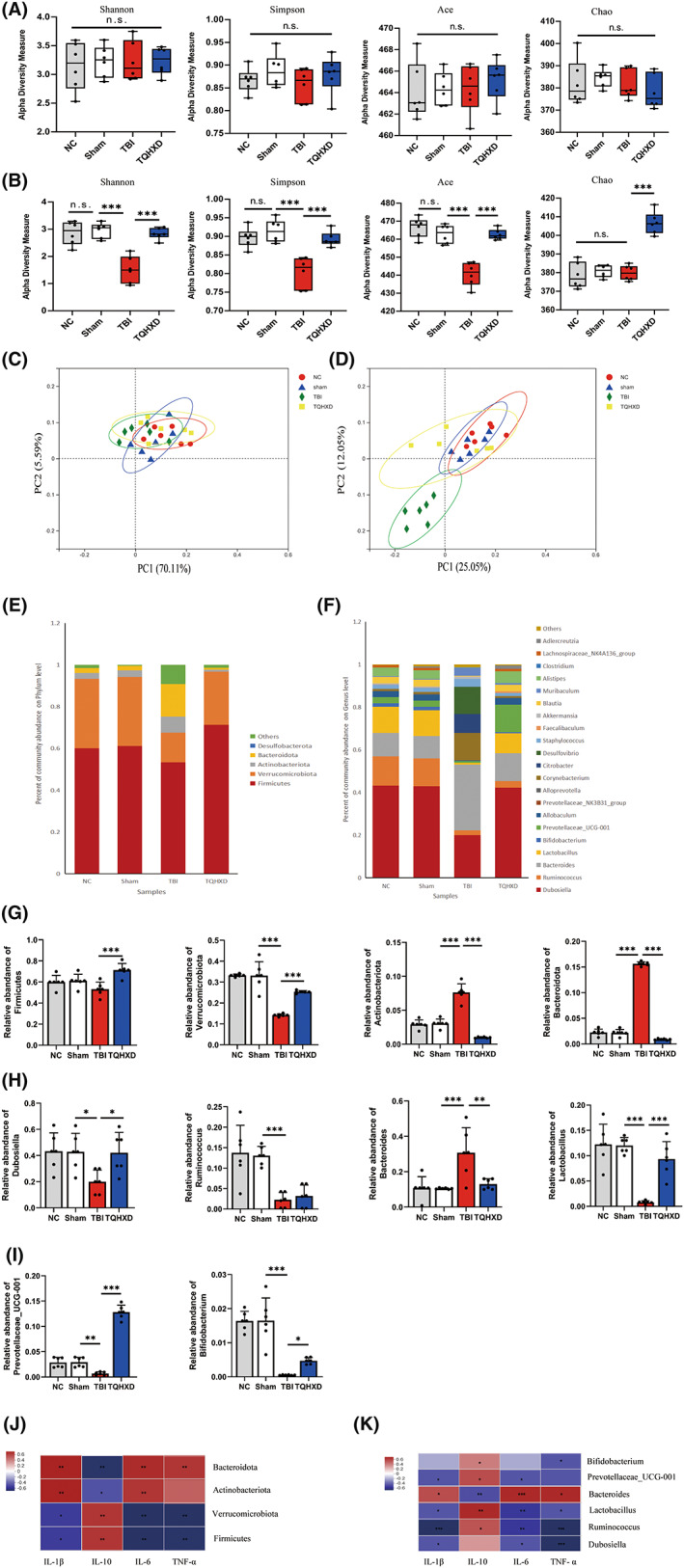
TQHXD relieved TBI‐induced inflammation in a microbiota‐dependent manner. (A) α‐diversity boxplot (Shannon, Simpson, ace, and Chao) on the 3rd day pre‐treatment and on the 3rd day post‐treatment (B). (C) PCoA of β‐diversity on the 3rd day pre‐treatment and on the 3rd day post‐treatment (D). (E) LDA score computed from features differently abundant among the NC, Sham, TBI and TQHXD groups at the phylum level and genus level (F). (G) Statistical analysis of the dominance bacterial communities in each group at the phylum level and genus level (H, I). (J) Spearman's rank test of association between inflammatory cytokines and bacteria at the phylum level and genus level (K). Data were presented as mean ± SD. *p < 0.05, **p < 0.01, ***p < 0.001, significantly different as indicated.

To determine whether intestinal microbiota mediated the therapeutic effects of TQHXD on TBI‐induced inflammation, we sequenced 16S ribosomal ribonucleic acid (rRNA) in fecal DNA extracted from the neutral control (NC), Sham, TBI, and TQHXD groups to investigate whether TQHXD altered the microbiome on Day 3 pre‐treatment and Day 3 post‐treatment. We detected gut microbial α‐diversity via the Shannon, Simpson, Abundance‐based Coverage Estimator (ACE), and Chao indices and found no significant differences among groups on Day 3 pre‐treatment (Figure 4A). On Day 3 post‐treatment, there was no significant difference between the NC and Sham groups, while the TQHXD group had greater α‐diversity than the TBI group (Figure 4B).

We also investigated bacterial‐community structure using β‐diversity analyses via principal‐coordinate analysis (PCoA) on Day 3 pre‐treatment and Day 3 post‐treatment. The obvious clustering distance among operational taxonomic units (OTUs) represented the different species compositions and structures among the four groups, demonstrating that these microbiomes differed in community structure. On Day 3 pre‐treatment, we saw no differences among the four groups (Figure 4C); on Day 3 post‐treatment, there was no significant difference between the NC and Sham groups, while compositions and structures in the TBI group were markedly different; these changes were partly reversed after TQHXD therapy (Figure 4D).

To further determine which bacteria were changed by TQHXD treatment on Day 3 after TBI, we performed high‐dimensional class comparisons via linear discriminant analysis (LDA) of effect size (LEfSe) to find differences in the dominance of bacterial communities at the phylum and genus levels in the NC, Sham, TBI, and TQHXD groups. At the phylum level, Firmicutes, Verrucomicrobiota, Actinobacteriota, and Bacteroidota were the predominant types of bacteria leading to intestinal‐microbiota dysbiosis (Figure 4E); at the genus level, *Dubosiella*, *Ruminococcus*, *Bacteroides*, *Lactobacillus*, *Bifidobacterium*, and *Prevotellaceae_UCG‐001* were the dominant types causing such dysbiosis (Figure 4F). Next, we conducted statistical difference analyses on these bacterial communities. We found that at the phylum level, TQHXD therapy dramatically elevated the relative abundance of beneficial bacteria, including *Firmicutes* and *Verrucomicrobiota*, and reduced the relative abundance of harmful ones, including Actinobacteriota and Bacteroidota (Figure 4G). At the genus level, beneficial bacteria such as *Dubosiella*, *Lactobacillus*, *Bifidobacterium*, *Ruminococcus*, and *Prevotellaceae_UCG‐001* displayed relatively lower abundance, and the harmful bacterium *Bacteroides* showed relatively higher abundance, in the TBI group. TQHXD therapy significantly reversed these trends except for that of *Ruminococcus* (Figure 4H,I).

Next, to investigate whether changes in inflammatory cytokines were associated with changes in gut microbiota, we conducted Spearman's rank test. At the phylum level, the anti‐inflammatory cytokine IL‐10 was associated positively with *Firmicutes* and *Verrucomicrobiota* and negatively with Actinobacteriota and Bacteroidota; pro‐inflammatory cytokines, including IL‐1β, IL‐6, and TNF‐α, correlated positively with *Actinobacteriota* and *Bacteroidota* and negatively with *Firmicutes* and *Verrucomicrobiota* (Figure 4J). At the genus level, IL‐10 level was positively associated with *Dubosiella*, *Lactobacillus*, *Bifidobacterium*, *Ruminococcus*, and *Prevotellaceae_UCG‐001* and negatively associated with *Bacteroides*. Levels of IL‐1β, IL‐6, and TNF‐α were positively associated with *Bacteroides* but negatively correlated with *Dubosiella*, *Lactobacillus*, *Bifidobacterium*, *Ruminococcus*, and *Prevotellaceae_UCG‐001* (Figure 4K). These results implied that the protective effect of TQHXD against TBI inflammation depended on altering intestinal‐microbiota diversity and composition.

### 3.4 TQHXD restored the epithelial and chemical barriers of the intestinal mucosal barrier

**FIGURE 5:**
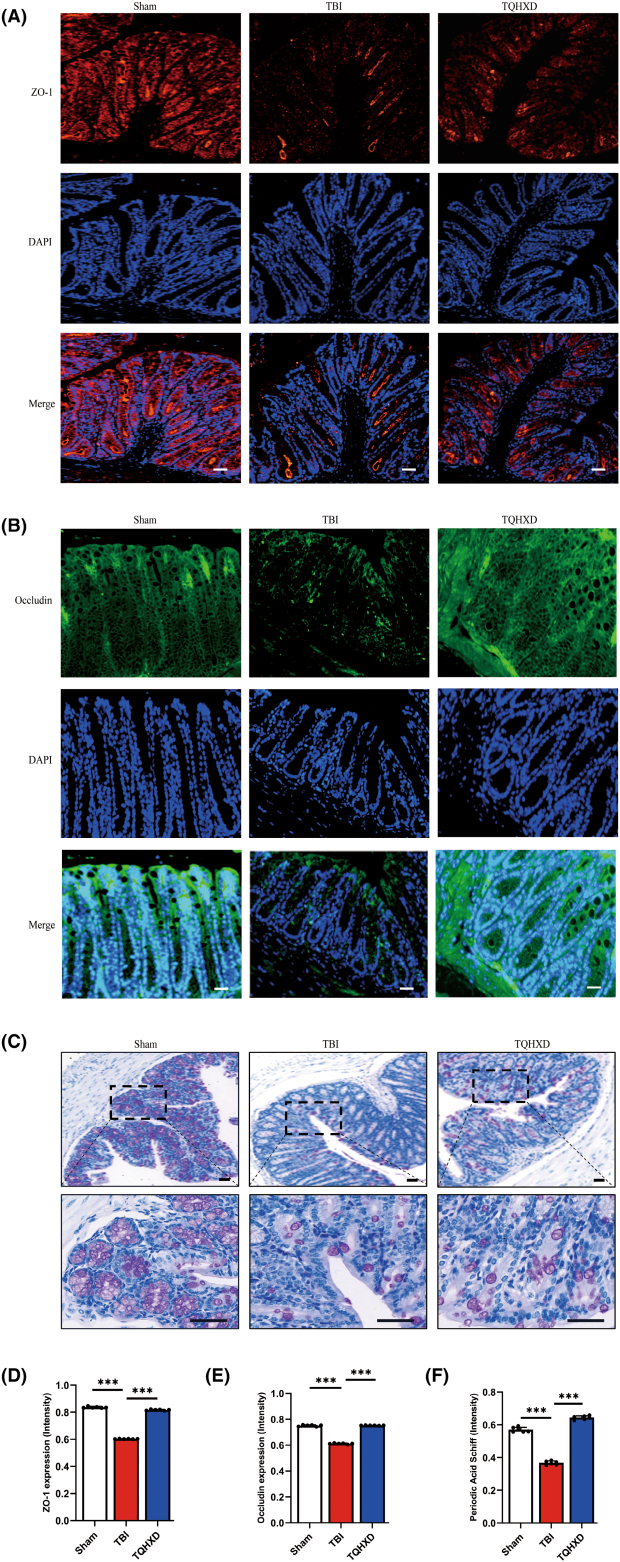
TQHXD restored the epithelial and chemical barriers of the intestinal mucosal barrier by promoting tight junctions and improving the function of goblet cell and associated mucus secretion. (A) Immunofluorescent staining of ZO‐1 and occludin (B). (C) PAS staining in colonic sections. (D) Quantitative analysis of ZO‐1, occludin (E) and mucus secretion (F). Data were presented as mean ± SD. *p < 0.05, **p < 0.01, ***p < 0.001, significantly different as indicated.

To verify the therapeutic effect of TQHXD on the epithelial and chemical barriers of the IMB in TBI‐induced IMB, we used IF and PAS staining to evaluate goblet cells and expression of tight‐junction proteins (TJPs), including ZO‐1 and occludin. As shown in Figure 5A,B, expression of ZO‐1 and occludin was visibly diminished in mice with TBI, whereas dissipation of these two proteins was partly repaired by TQHXD (Figure 5A,B,D,E). Similarly, via PAS staining we confirmed a severe reduction in goblet cells and, correspondingly, less mucus secreted by these cells after TBI compared with the Sham group. Importantly, TQHXD treatment dramatically reversed the decrease in goblet cells and associated mucus (Figure 5C,F). In summary, our data implied that TQHXD could restore the epithelial and chemical barriers of the IMB in mice with TBI‐induced intestinal inflammation.

### 3.5 TQHXD regulated expression of CD36/15‐LO/NR4A1 in colon tissue of mice with TBI‐induced intestinal inflammation and mucosal barrier damage

**FIGURE 6:**
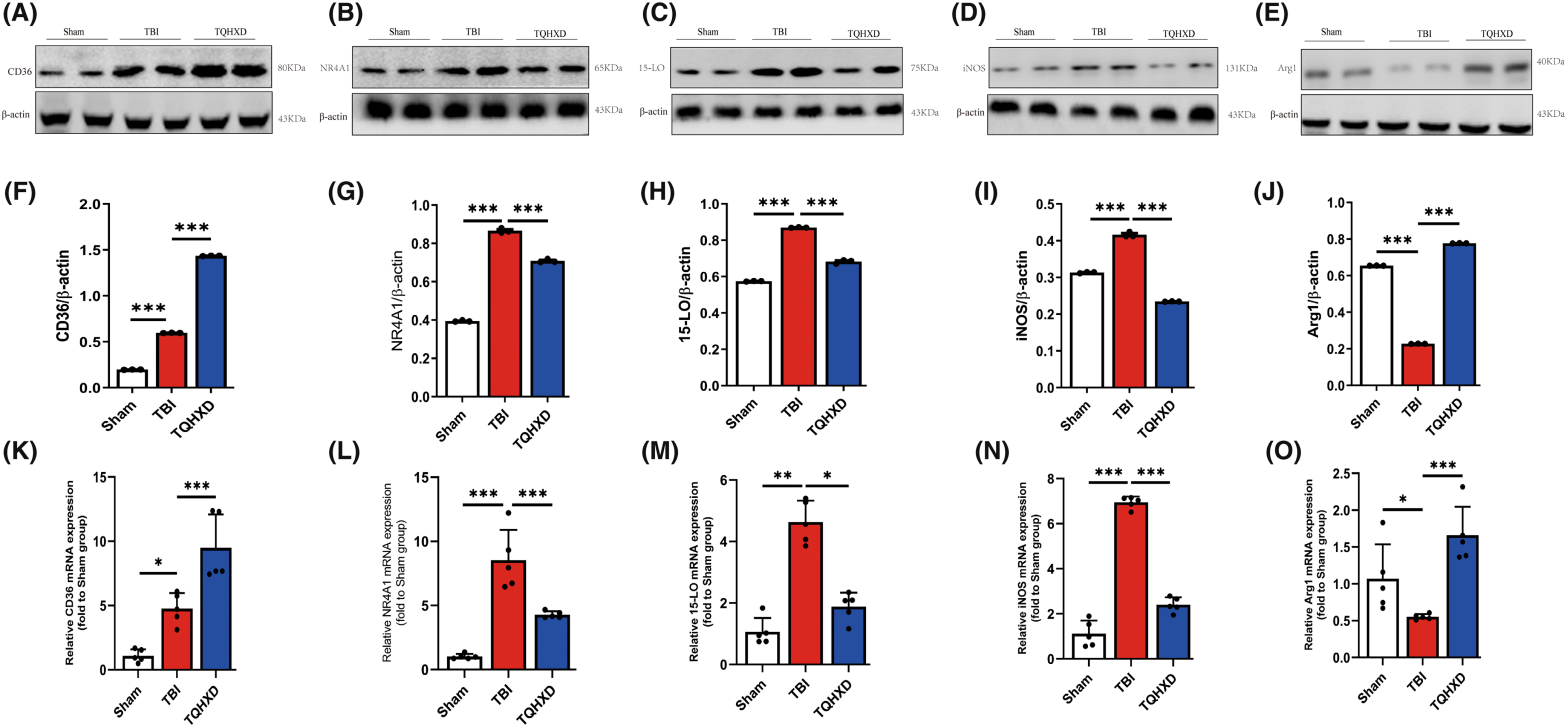
TQHXD regulated expression of CD36/15‐LO/NR4A1 in colon tissue of mice with TBI‐induced intestinal inflammation and mucosal‐barrier damage. Immunoblotting analysis for CD36 (A), NR4A1 (B), 15LO (C), iNOS (D), Arg1 (E). Quantitative analysis of CD36 (F), NR4A1 (G), 15‐LO (H), iNOS (I), Arg1 (J). And mRNAs expression of CD36 (K), NR4A1 (L), 15‐LO (M), iNOS (N), Arg1 (O) were detected by RT‐PCR. Data were presented as mean ± SD. *p < 0.05, **p < 0.01, ***p < 0.001, significantly different as indicated.

Our previous study confirmed that CD36/15‐LO/NR4A1 signaling regulates brain function recovery in mice with TBI,^17^ but this finding had not been clarified in a model of TBI‐induced intestinal inflammation and IMB damage. Therefore, we used Western blot (WB) to detect CD36 levels in colon tissue. We found that expression thereof was markedly increased in the TBI group and that this trend was further enhanced after oral administration of TQHXD (Figure 6A,F). In addition, expression of 15‐LO and NR4A1 in colon tissue was remarkably elevated, and TQHXD significantly suppressed increases of such expression caused by TBI (Figure 6B,C,G,H). We verified those results using RT‐PCR (Figure 6K–M). Taken together, these results strongly demonstrated that TQHXD could relieve intestinal inflammation and IMB damage by stimulating CD36/15‐LO/NR4A1 signaling.

### 3.6 CD36 deficiency aggravated TBI‐induced intestinal inflammation and mucosal barrier damage, which TQHXD could not rescue

**FIGURE 7:**
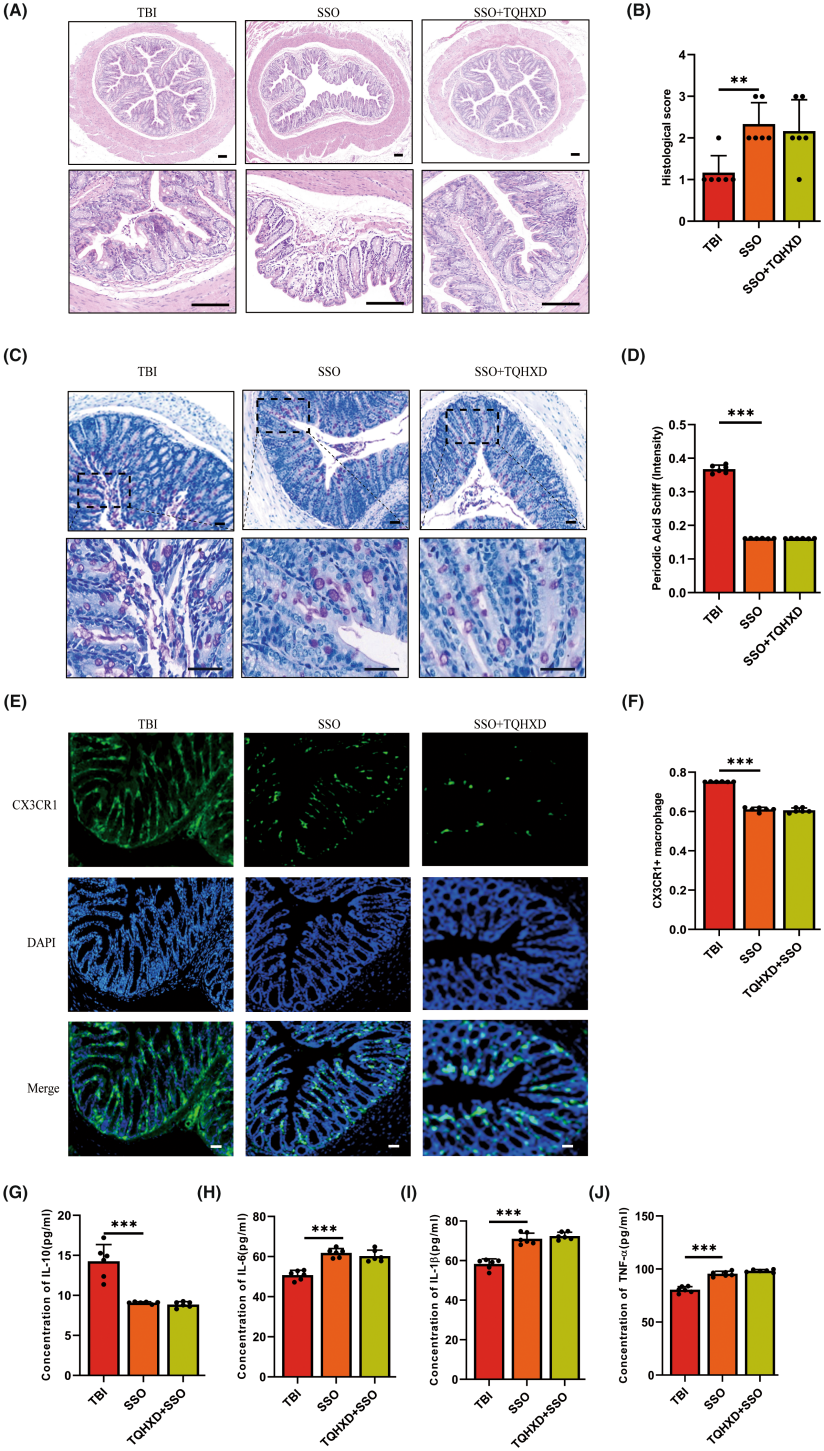
CD36 deficiency aggravated TBI‐induced intestinal inflammation and barrier damage that could not be rescued with TQHXD. (A) HE staining of colon tissue (magnification above ×40, magnification below ×200). (B) Histological scores of colon tissue. (C, D) PAS staining in colonic sections. (E) The expression of green fluorescent protein of CX3CR1 on macrophages (magnification ×200) and its quantitative analysis (F). The levels of cytokines including IL‐10 (G), IL‐6 (H), IL‐1β (I) and TNF‐α (J). Data were presented as mean ± SD. *p < 0.05, **p < 0.01, ***p < 0.001, significantly different as indicated.

**FIGURE 8:**
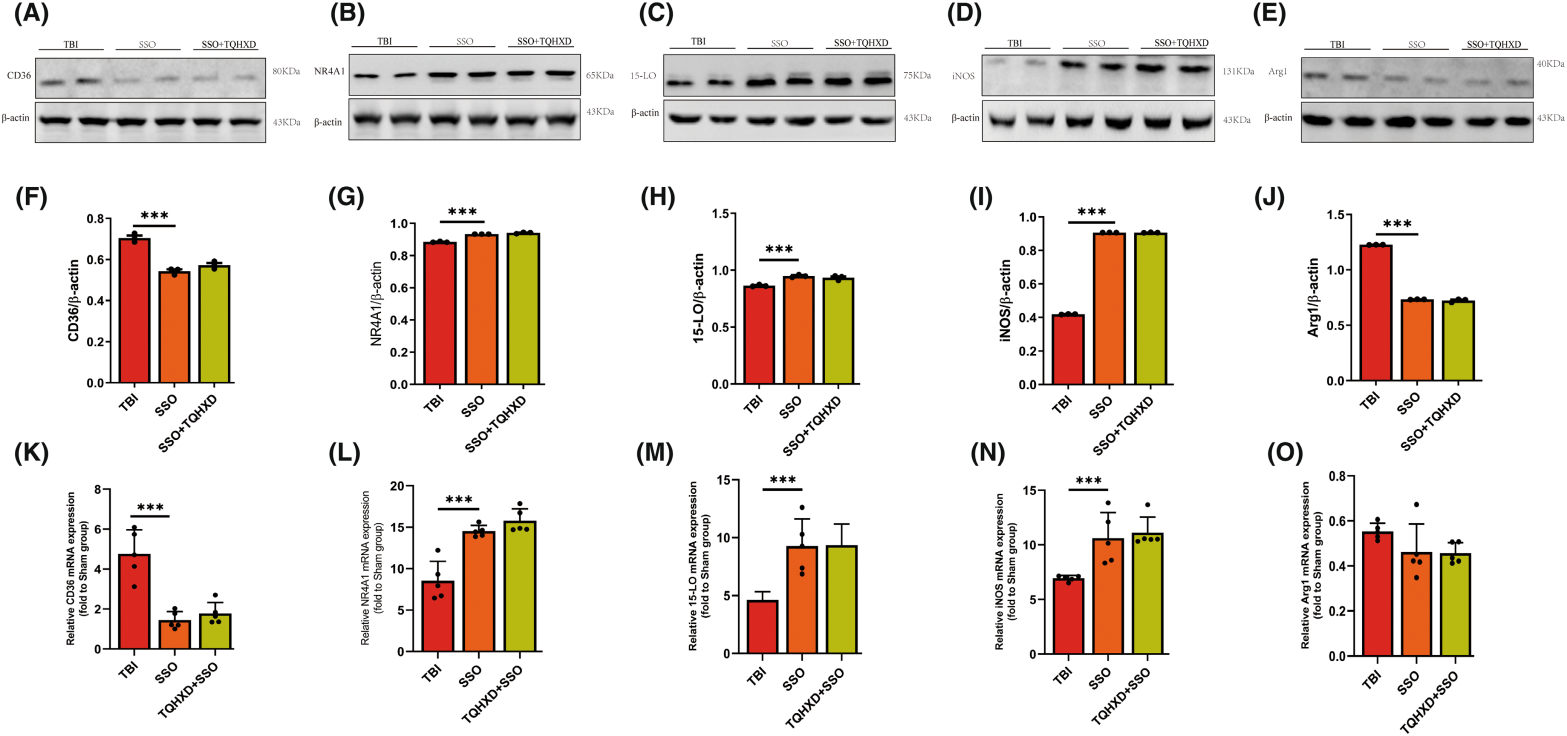
TQHXD ameliorated TBI‐induced GI dysfunction by regulating CD36/15‐LO/NR4A1 signaling, which failed when CD36 were deficient. Immunoblotting analysis for CD36 (A), NR4A1 (B), 15LO (C), iNOS (D), Arg1 (E). Quantitative analysis of CD36 (F), NR4A1 (G), 15‐LO (H), iNOS (I), Arg1 (J). And mRNAs expression of CD36 (K), NR4A1 (L), 15‐LO (M), iNOS (N), Arg1 (O) were detected by RT‐PCR. Data were presented as mean ± SD. *p < 0.05, **p < 0.01, ***p < 0.001, significantly different as indicated.

To further verify whether TQHXD exerted protective effects by activating CD36, we applied SSO, a CD36 inhibitor that can bind to CD36 receptors on the surfaces of macrophages, to treat mice with TBI. As expected, H&E staining showed that reduced goblet cells, disordered glandular structure, and infiltration of inflammatory cells caused by TBI were further aggravated in mice orally gavaged with SSO, and TQHXD could not rescue these changes (Figure 7A,B). In addition, CD36 deficiency caused a remarkable further reduction in intestinal goblet cells and associated mucus secretion, which TQHXD could not reverse (Figure 7C,D). As shown in Figure 7E,F, the CX3CR1^+^ macrophage count was reduced in the SSO group and not elevated in the TQHXD group. Similarly, CD36 deficiency resulted in a profound reduction in IL‐10 and an increase in IL‐6, IL‐1β, and TNF‐α, which TQHXD therapy could not resolve (Figure 7G–J). Importantly, CD36 levels were visibly reduced and expression of 15‐LO and NR4A1 markedly increased in mice orally gavaged with SSO, which could not be reversed by TQHXD (Figure 8A–C,F–H). We verified this result using RT‐PCR (Figure 8K–M). Taken together, these results showed that CD36 deficiency aggravated TBI‐induced intestinal inflammation and IMB damage, and this change could not be rescued by TQHXD.

### 3.7 TQHXD regulated innate and adaptive immune homeostasis of TBI‐induced colitis but could not exert a protective effect in the absence of CD36

**FIGURE 9:**
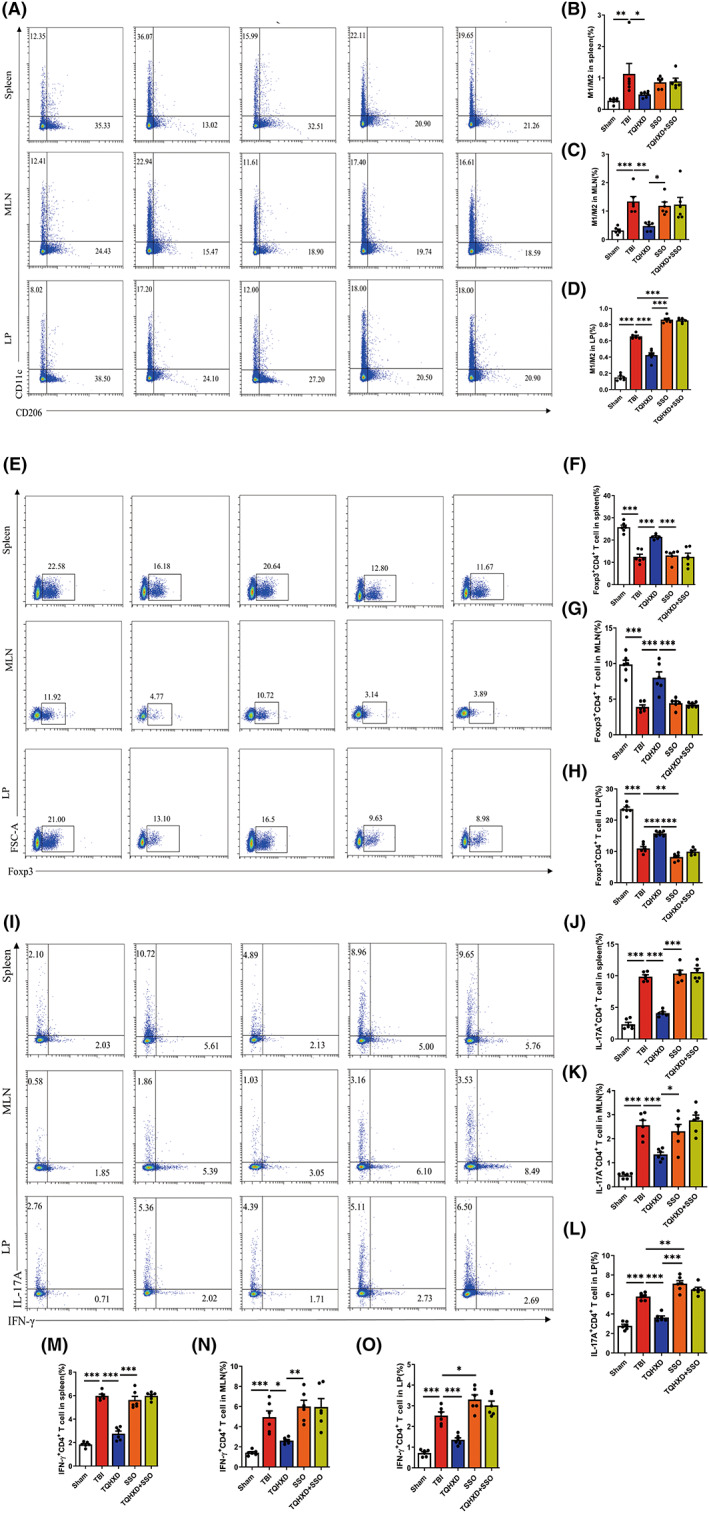
TQHXD regulated innate and adaptive immune homeostasis of TBI‐induced colitis but could not exert a protective effect in the absence of CD36. (A) M1/M2 ratio of macrophage in spleen, MLN, and LP were analyzed by flow cytometry and the bar charts of statistical analysis of spleen (B), MLN (C), and LP (D). (E) Foxp3^+^CD4^+^ (Treg) cells in spleen, MLN and LP and the bar charts of statistical analysis of spleen (F), MLN (G) and LP (H). (I) IFN‐γ^+^CD4^+^ T cells and IL‐17A^+^CD4^+^ T cells in spleen, MLN and LP were analyzed by flow cytometry, and the bar charts of statistical analysis (J–O). Data were presented as mean ± SD. *p < 0.05, **p < 0.01, ***p < 0.001, significantly different as indicated.

To explore TQHXD's modulatory effect on intestinal immune homeostasis, we used flow cytometry (FCM) to detect phenotypes of macrophages and lymphocytes in the spleen, MLNs, and colonic LP in each group. The data showed that M1/M2 macrophage ratios in cells isolated from the spleens, MLNs, and LPs of TBI‐induced mice were dramatically enhanced compared with those of mice in the Sham group, while TQHXD significantly decreased these ratios. After SSO administration, M1/M2 macrophage ratios in LP were further enhanced in comparison with the TBI group and could not be rescued by TQHXD treatment (Figure 9A–D). We used WB and RT‐PCR to measure levels of iNOS, the phenotypic signature of M1, and ARG1, a specific marker of M2 macrophages. Consistently, protein and messenger RNA (mRNA) levels of iNOS were significantly elevated in the TBI group and inhibited in the TQHXD group (Figure 6D,I,N). Protein and mRNA expression of ARG1 was also remarkably decreased by TBI, and this reduction was significantly suppressed by TQHXD treatment (Figure 6E,J,O). However, TQHXD could not alter these trends after the oral administration of SSO. Additionally, Foxp3^+^CD4^+^ T‐cell counts in spleen, MLN, and LP cells were upregulated (Figure 9E–H), while those of IL‐17A^+^CD4^+^ and IFN‐γ^+^CD4^+^ T cells were downregulated, in the TQHXD group. However, TQHXD administration could not reverse these trends in LP tissue after SSO treatment (Figure 9I–O). Taken together, these data indicated that TQHXD could regulate immune homeostasis in TBI‐induced colitis by modulating the M1/M2 ratios of macrophages and restoring the balance of Th_17_/Tregs and Th_1_/Tregs, but it could not exert these protective effects in the absence of CD36.

### 3.8 CX3CR1 deficiency exacerbated TBI‐induced colitis, which could not be rescued with TQHXD and was further aggravated by SSO administration

**FIGURE 10:**
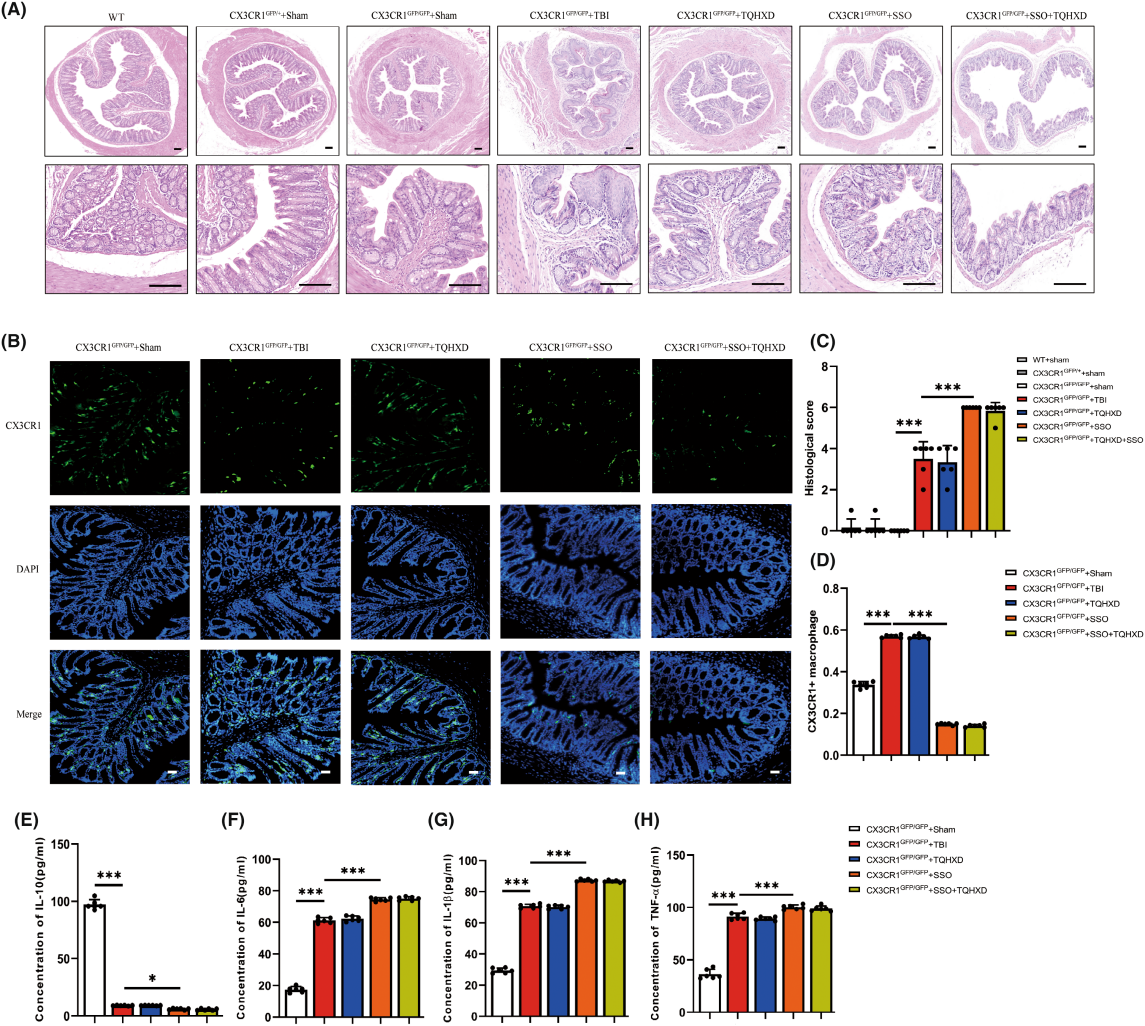
CX3CR1 deficiency exacerbated TBI‐induced colitis, which could not be rescued with TQHXD and was further aggravated by SSO administration. (A) HE staining of colon tissue (magnification above ×40, magnification below ×200). (B) The expression of green fluorescent protein of CX3CR1 on macrophages (magnification ×200). (C) Histological score of colon tissue. (D) The quantitative analyses of Figure 10B. The levels of cytokines including IL‐10 (E), IL‐6 (F), IL‐1β (G), and TNF‐α (H). Data were presented as mean ± SD. *p < 0.05, **p < 0.01, ***p < 0.001, significantly different as indicated

To verify whether TQHXD protected the gut from inflammation via CX3CR1, we subjected CX3CR1^GFP^^/+^ and CX3CR1^GFP^ ^/GFP^ reporter mice, in which CX3CR1 is replaced with GFP on macrophages in one and two alleles, respectively, to a TBI‐induced colitis model. We compared H&E staining results in the wild‐type (WT) + Sham, CX3CR1^GFP^^/+^ + Sham, and CX3CR1^GFP^ ^/GFP^ + Sham groups and found inconspicuous pathological features. Compared with the CX3CR1^GFP^ ^/GFP^ + Sham group, in the CX3CR1^GFP^ ^/GFP^ + TBI group we observed fewer goblet cells, disordered glandular structure, and infiltration of inflammatory cells; these changes could not be rescued with TQHXD and were further aggravated by SSO administration (Figure 10A,C). IF staining showed that TQHXD treatment could not increase the number of CX3CR1^+^ macrophages; moreover, this number was further reduced after SSO administration (Figure 10B,D). Similarly, in comparison with the CX3CR1^GFP^ ^/GFP^ + TBI group, TQHXD treatment could not upregulate levels of IL‐10 or downregulate those of IL‐1β, IL‐6, and TNF‐α in colon tissues in the CX3CR1^GFP^ ^/GFP^ + TQHXD group; these trends were further worsened following SSO therapy (Figure 10E **–**H). These data indicated that CX3CR1 deficiency exacerbated TBI‐induced colitis, and this exacerbation could not be rescued with TQHXD but was further aggravated by SSO administration.

### 3.9 CX3CR1 deficiency nullified the therapeutic effect of TQHXD via disruption of intestinal‐microbiome homeostasis

**FIGURE 11:**
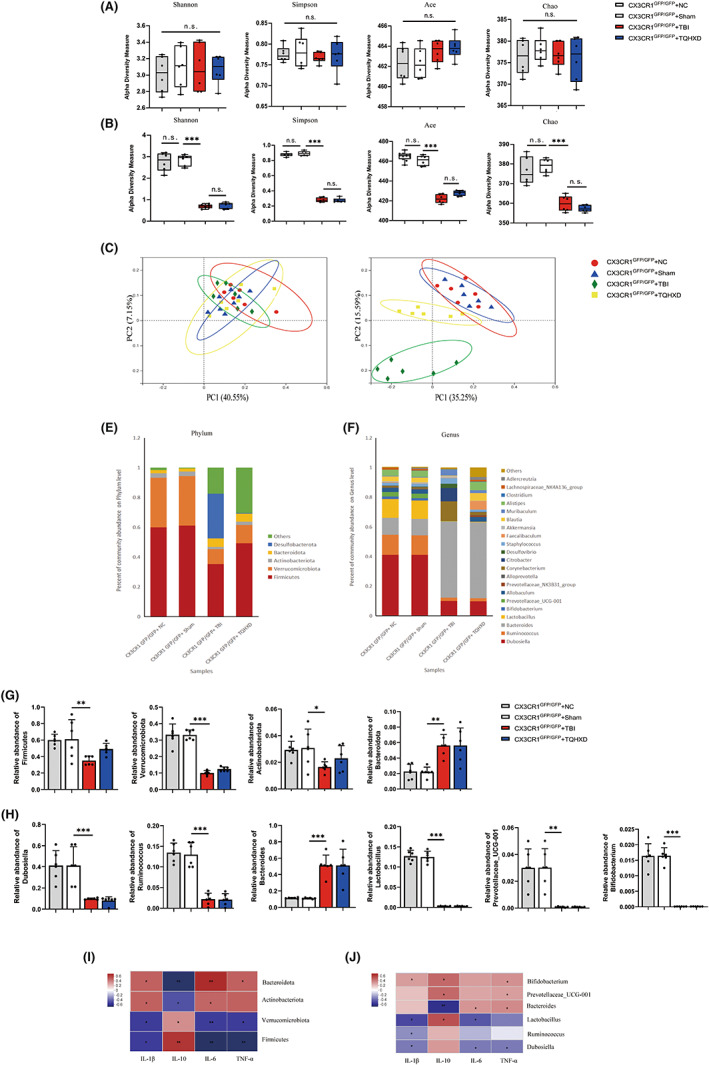
CX3CR1 deficiency nullified the therapeutic effect of TQHXD via disruption of intestinal‐microbiome homeostasis. (A) α‐diversity boxplot (Shannon, Simpson, ace, and Chao) on the 3rd day pre‐treatment and on the 3rd day post‐treatment (B). (C) PCoA of β‐diversity on the 3rd day pre‐treatment and on the 3rd day post‐treatment (D). (E) LDA score computed from features differently abundant among the NC, Sham, TBI and TQHXD groups at the phylum level and genus level (F). (G) Statistical analysis of the dominance bacterial communities in each group at the phylum level and genus level (H). (I) Spearman's rank test of association between inflammatory cytokines and bacteria at the phylum level and genus level (J). Data were presented as mean ± SD. *p < 0.05, **p < 0.01, ***p < 0.001, significantly different as indicated.

The Shannon, Simpson, ACE, and Chao indices showed no significant differences among groups on Day 3 pre‐treatment (Figure 11A), nor did they find any significant difference between the CX3CR1^GFP/GFP^ + TQHXD group and the CX3CR1^GFP/GFP^ + TBI group on Day 3 post‐treatment (Figure 11B). Similarly, β‐diversity analyses indicated no significant differences on Day 3 pre‐treatment (Figure 11C). Compared with the CX3CR1^GFP/GFP^ + Sham group, β‐diversity values in mice with TBI were different on Day 3 post‐treatment; nevertheless, TQHXD could not alter this trend (Figure 11D).

Next, we further clarified which bacteria were changed at Day 3 post‐treatment in CX3CR1^GFP/GFP^ mice with TBI. At the phylum level, *Firmicutes*, *Verrucomicrobiota*, *Actinobacteriota*, and *Bacteroidota* were the majority types of bacteria resulting in intestinal‐microbiota dysbiosis in the four groups (Figure 11E); at the genus level, *Dubosiella*, *Ruminococcus*, *Bacteroides*, *Lactobacillus*, *Bifidobacterium* and *Prevotellaceae_UCG‐001* were the dominant types causing such dysbiosis (Figure 11F). Compared with the CX3CR1^GFP/GFP^ + TBI group, the reduction at the phylum level in the relative abundance of bacteria including *Firmicutes* and *Verrucomicrobiota*, and the increase in same of *Bacteroidota*, could not be rescued in the CX3CR1^GFP/GFP^ + TQHXD group (Figure 11G). Notably, Actinobacteriota, a phylum of harmful bacteria, was less abundant in the CX3CR1^GFP/GFP^ + TBI group, indicating that CX3CR1 knockout (KO) mice had a certain self‐repair capability that promoted the balance of gut microbes after TBI. Consistently, at the genus level, the decline in the relative abundance of beneficial bacteria, including *Dubosiella*, *Lactobacillus*, *Bifidobacterium*, *Ruminococcus*, and *Prevotellaceae_UCG‐001*, and the increased relative abundance of the harmful *Bacteroides*, in the CX3CR1^GFP/GFP^ + TBI group was not also altered in comparison with the CX3CR1^GFP/GFP^ + TQHXD group (Figure 11H).

Then, we used Spearman's rank test to explore whether inflammatory cytokine changes were associated with changes in gut microbiota. At the phylum level, IL‐10 levels were associated positively with *Firmicutes* and *Verrucomicrobiota* and negatively with *Actinobacteriota* and *Bacteroidota*; levels of IL‐1β, IL‐6, and TNF‐α correlated positively with *Actinobacteriota* and *Bacteroidota* but negatively with *Firmicutes* and *Verrucomicrobiota* (Figure 11I). At the genus level, IL‐10 levels were positively associated with *Dubosiella*, *Lactobacillus*, *Bifidobacterium*, *Ruminococcus* and *Prevotellaceae_UCG‐001* but negatively associated with *Bacteroides*, while levels of IL‐1β, IL‐6, and TNF‐α were positively associated with *Bacteroides* but showed the opposite trend for *Dubosiella*, *Lactobacillus*, *Bifidobacterium*, *Ruminococcus*, and *Prevotellaceae_UCG‐001*. Overall, these data implied that the intestinal inflammatory reaction induced by TBI depended on altering intestinal‐microbiota diversity and composition (Figure 11J). These data confirmed that CX3CR1 deficiency nullified the therapeutic effect of TQHXD via disruption of intestinal‐microbiome homeostasis.

### 3.10 CX3CR1 insufficiency eliminated the therapeutic effect of TQHXD by damaging the epithelial and chemical barriers of the intestinal mucosal barrier, and such damage worsened after SSO treatment

**FIGURE 12:**
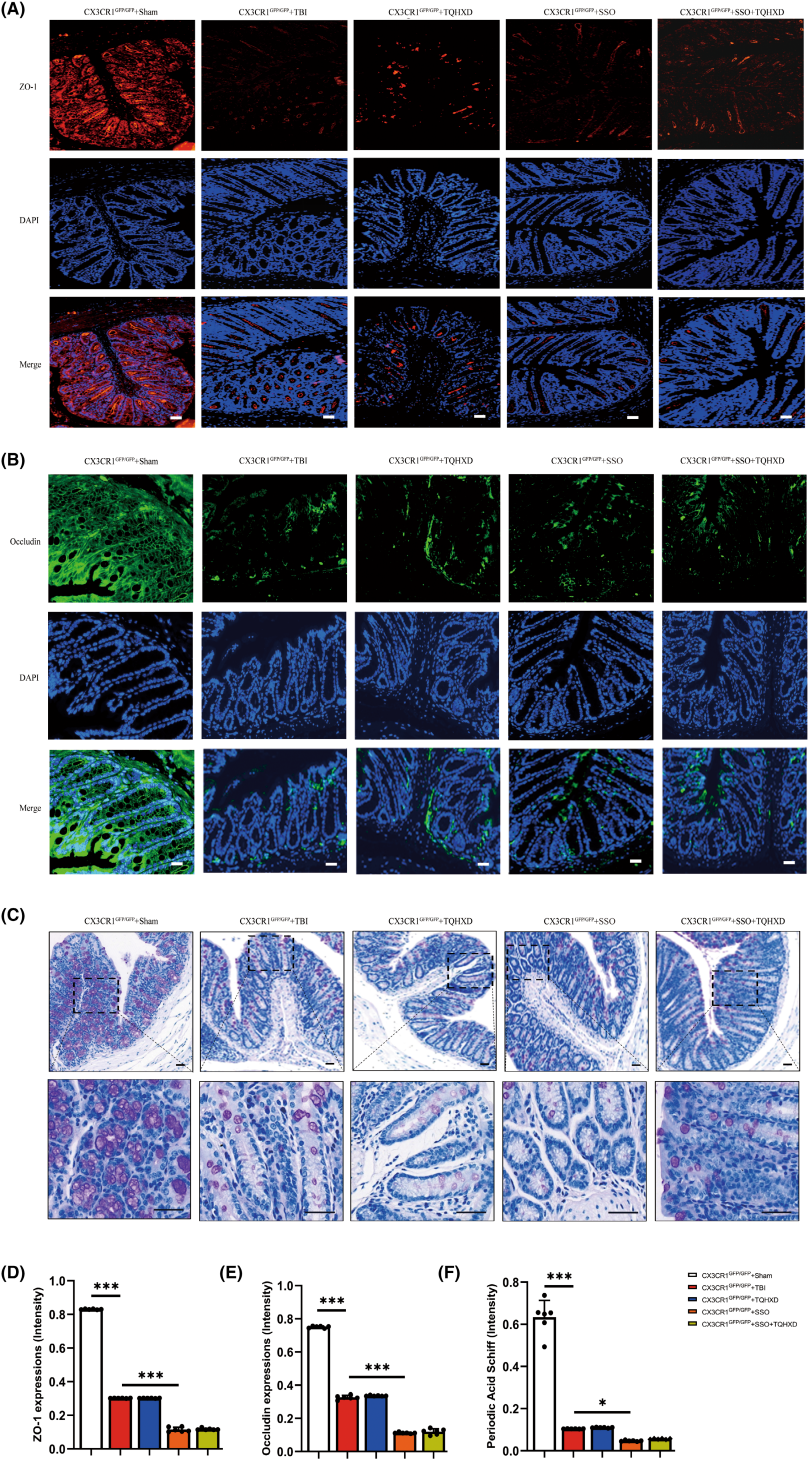
CX3CR1 insufficiency eliminated the therapeutic effect of TQHXD by damaging the epithelial and chemical barriers of the intestinal mucosal barrier, and such damage worsened after SSO treatment. (A) Immunofluorescent staining of ZO‐1 and occludin (B). (C) PAS staining in colonic sections. (D) quantitative analysis of ZO‐1, occludin (E) and mucus secretion (F). Data were presented as mean ± SD. *p < 0.05, **p < 0.01, ***p < 0.001, significantly different as indicated.

To understand whether the therapeutic effects of TQHXD depended on the integrity and function of the IMB's epithelial and chemical barriers in CX3CR1 KO mice, we performed IF and PAS staining. As shown in Figure 12A,B,D,E, levels of ZO‐1 and occludin were visibly reduced in the CX3CR1^GFP/GFP^ + TBI group, and this reduction was not rescued after TQHXD administration. Moreover, SSO treatment aggravated the suppression of ZO‐1 and occludin expression. TQHXD therapy could not rescue the decrease in goblet cells and associated mucus, and SSO treatment worsened this decrease in CX3CR1^GFP/GFP^ mice with TBI (Figure 12C,F). These data demonstrated that CX3CR1 insufficiency eliminated the therapeutic effect of TQHXD by damaging the epithelial and chemical barriers of the IMB, and this damage was worsened after SSO treatment.

### 3.11 CX3CR1 insufficiency eradicated the protective effect exerted by TQHXD via regulation of innate and adaptive immune homeostasis, and this eradication was exacerbated after SSO treatment

**FIGURE 13:**
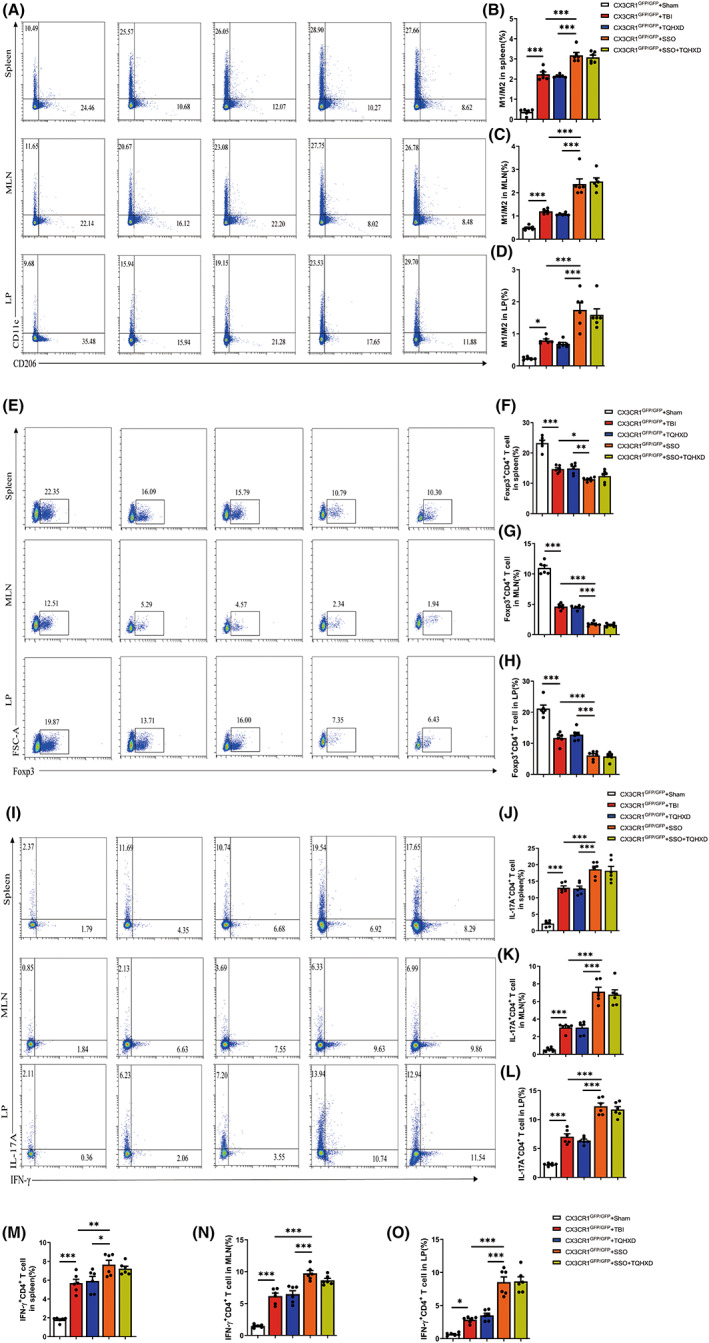
CX3CR1 insufficiency eradicated the protective effect exerted by TQHXD via regulation of innate and adaptive immune homeostasis, and this eradication was exacerbated after SSO treatment. (A) M1/M2 ratio of macrophage in spleen, MLN and LP were analyzed by flow cytometry and the bar charts of statistical analysis of spleen (B), MLN (C) and LP (D). (E) Foxp3^+^CD4^+^ (Treg) cells in spleen, MLN, and LP and the bar charts of statistical analysis of spleen (F), MLN (G) and LP (H). (I) IFN‐γ^+^CD4^+^ T cells and IL‐17A^+^CD4^+^ T cells in spleen, MLN and LP were analyzed by flow cytometry, and the bar charts of statistical analysis (J–O). Data were presented as mean ± SD. *p < 0.05, **p < 0.01, ***p < 0.001, significantly different as indicated.

**FIGURE 14:**
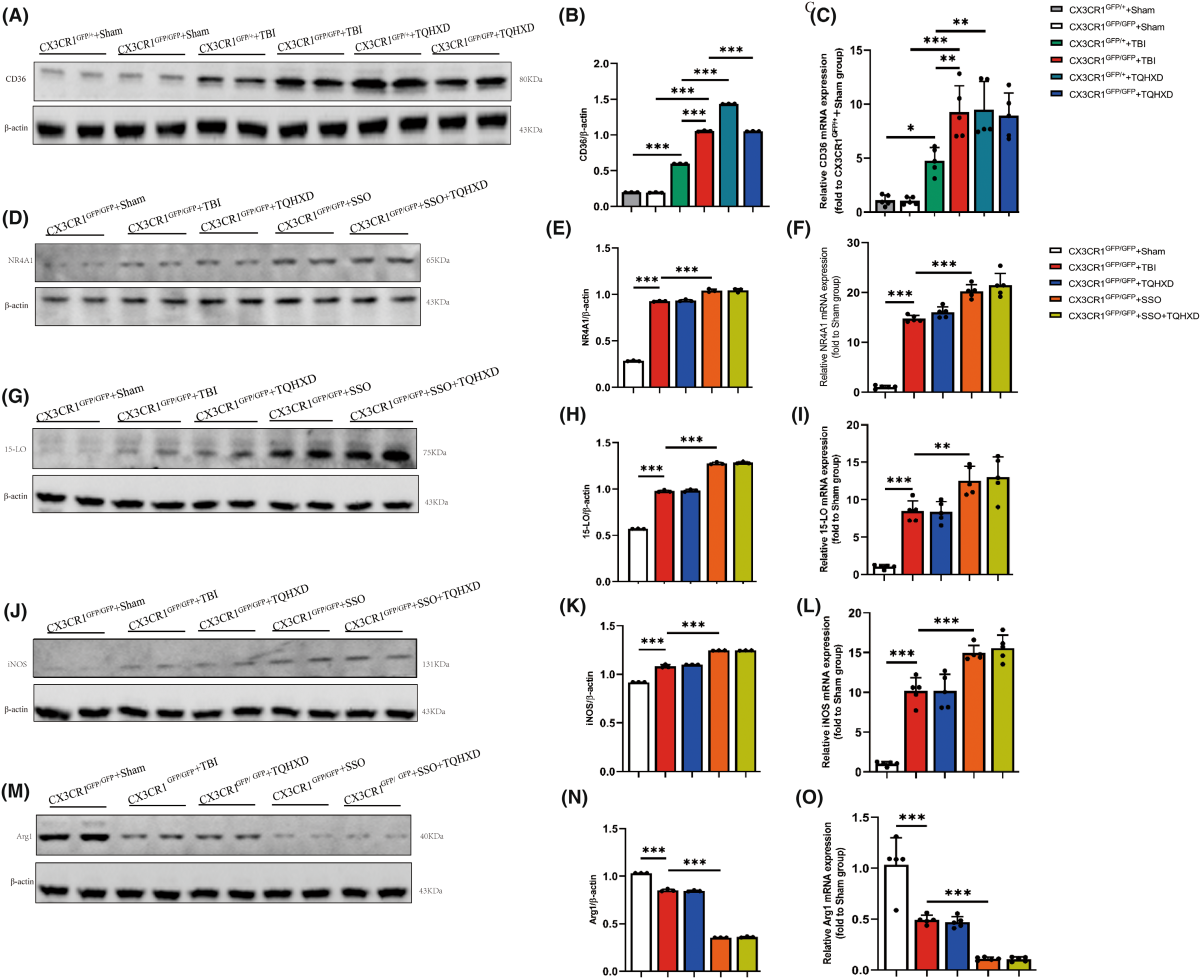
TQHXD eradicated TQHXD's protective effects in TBI‐induced colitis through CD36/15‐LO/NR4A1 signaling in CX3CR1 knockout mice. Immunoblotting analysis for CD36 (A), NR4A1 (B), 15LO (C), iNOS (D), Arg1 (E). Quantitative analysis of CD36 (F), NR4A1 (G), 15‐LO (H), iNOS (I), Arg1 (J). And mRNAs expression of CD36 (K), NR4A1 (L), 15‐LO (M), iNOS (N), Arg1 (O) were detected by RT‐PCR. Data were presented as mean ± SD. *p < 0.05, **p < 0.01, ***p < 0.001, significantly different as indicated.

Compared with the CX3CR1^GFP/GFP^ + TBI group, we saw no significant change in M1/M2 ratios of macrophages in the CX3CR1^GFP/GFP^ + TQHXD group; this was exacerbated in spleen, MLN, and LP cells of the CX3CR1^GFP/GFP^ + SSO group (Figure 13A–D). In addition, TQHXD failed to reduce iNOS levels or to increase ARG1 expression; meanwhile, SSO worsened the reduction of iNOS and elevation of ARG1 in colon tissue of CX3CR1^GFP/GFP^ mice with TBI (Figure 14J–O). Similarly, TQHXD not only failed to increase counts of Foxp3^+^CD4^+^ T cells but also could not decrease those of IL‐17A^+^CD4^+^ or IFN‐γ^+^CD4^+^ T cells in spleen, MLN, and LP cells of CX3CR1^GFP/GFP^ mice with TBI. SSO further intensified this reduction in Foxp3^+^CD4^+^ T cells and increase in IL‐17A^+^CD4^+^ and IFN‐γ^+^CD4^+^ T cells in CX3CR1^GFP/GFP^ mice with TBI (Figure 13E–O). Taken together, these data showed that CX3CR1 insufficiency eradicated the protective effects of TQHXD via the regulation of innate and adaptive immune homeostasis, and this eradication was exacerbated after SSO treatment.

### 3.12 TQHXD eradicated TQHXD's protective effects in TBI‐induced colitis through CD36/15‐LO/NR4A1 signaling in CX3CR1 knockout mice

Most importantly, we aimed to show whether TQHXD exerted therapeutic effects by stimulating CX3CR1 and CD36/15‐LO/NR4A1 signaling. The results demonstrated that the level of CD36 in the CX3CR1^GFP/GFP^ + TBI group was increased in comparison with the CX3CR1^GFP/+^ + Sham group. Compared with the CX3CR1^GFP/+^ + TBI group, TQHXD exerted therapeutic effects in the CX3CR1^GFP/+^ + TQHXD group, while CX3CR1 insufficiency eradicated such protective effects in the CX3CR1^GFP/GFP^ + TQHXD group (Figure 14A–C). In the absence of CD36, levels of NR4A1 and 15‐LO proteins were elevated, in comparison with CX3CR1^GFP/GFP^ mice with TBI, and TQHXD failed to rescue these changes (Figure 14D–I). These data indicated that TQHXD could exert protective effects in mice with TBI‐induced colitis by stimulating CX3CR1/CD36/15‐LO/NR4A1 signaling.

## 4 DISCUSSION

GI dysfunction, a common peripheral‐organ complication after TBI,^4^ is characterized by gut inflammation and destruction of the IMB. The clinical presentations are sudden abdominal pain and loose and/or bloody stools. TQHXD, a typical prescription with marked anti‐inflammatory and anti‐OS effects on brain injury, has been used clinically as a traditional Chinese medicine for more than 1000 years due to its strong effectiveness in treating brain disease‐induced gut injury.^22^, ^31^ In this study, we comprehensively illustrated the mechanisms by which TQHXD ameliorated TBI‐induced GI dysfunction, its protective action on gut inflammation and IMB disruption, and the signaling it depends on.

### 4.1 TQHXD relieved TBI‐induced inflammation in a microbiota‐dependent manner, which was eliminated when CX3CR1 was insufficient

Clinical and animal studies have shown that the composition of intestinal microbiota is visibly distinct between healthy individuals and those with TBI.^32^, ^33^ A recent study demonstrated that antibiotic administration prior to TBI relieved TBI‐induced gut inflammation and IMB disruption.^34^ Reduction in microbial abundance and dysbiosis of harmful versus probiotic bacteria play crucial roles in TBI.^35^ Therefore, the homeostasis of intestinal microbiota must be maintained in TBI therapy. In our study, the Shannon, Simpson, ACE, and Chao α‐diversity indices, as well as the β‐diversity index of PCoA, confirmed that prior to Day 3 of TBI, no changes occurred in intestinal microbiota. On Day 3 after TBI, α‐diversity indices showed that TQHXD‐treated mice were more diverse than those in the TBI group; analysis of β‐diversity via PCoA revealed that mice in the TQHXD group had obvious clustering separation from those in the TBI group, implying that TQHXD treatment remarkably altered biological‐community structures. Further analysis by LEfSe of the NC, Sham, TBI, and TQHXD groups revealed that TQHXD could change the dominance of bacterial communities, that is, it elevated the previously reduced levels of beneficial bacteria and reduced the formerly increased levels of harmful bacteria. At the phylum level, such beneficial bacteria as *Firmicutes* and *Verrucomicrobiota* were more abundant, and harmful bacteria such as Actinobacteriota and Bacteroidota less so, in the TQHXD group. At the genus level, probiotic bacteria including *Dubosiella*, *Ruminococcus*, *Lactobacillus*, *Bifidobacterium*, and *Prevotellaceae_UCG‐001* showed higher abundance and *Bacteroides* showed lower abundance in the TQHXD group. Nevertheless, the therapeutic effect of TQHXD disappeared when CX3CR1 was deficient. Interestingly, Actinobacteriota, a phylum of harmful bacteria, was less abundant in the CX3CR1^GFP/GFP^ + TBI group, which indicated that CX3CR1 KO mice had a certain self‐repair capability that promoted a balance of gut microbes after TBI. It is worth noting that anti‐inflammatory cytokines, including IL‐10, were positively associated with probiotic bacteria and negatively associated with pathogenic bacteria; the opposite was true of pro‐inflammatory cytokines such as IL‐1β, IL‐6, and TNF‐α. These associations were weaker in CX3CR1 KO mice. These results implied that the protective effects of TQHXD on TBI‐induced colitis depended on altering intestinal‐microbiota diversity and composition to reduce the inflammatory response. Mao and Yi et al.^36^, ^37^, ^38^, ^39^ verified that probiotic bacteria, including *Firmicutes*, *Verrucomicrobiota*, *Dubosiella*, *Ruminococcus*, *Lactobacillus*, *Bifidobacterium*, and *Prevotellaceae_UCG‐001*, were associated with improvements in diseases of the GI system due to increases in the secretion of IL‐10; this was consistent with our findings. Moreover, Hua et al. and Peng et al.^38^, ^40^ proved that harmful bacteria, including *Actinobacteriota*, *Bacteroidota*, and *Bacteroides*, were related to the severity of gut barrier disruption via increases in IL‐1β, IL‐6, and TNF‐α, which revealed the therapeutic action of TQHXD via modulation of the IMB's biological barrier.

### 4.2 TQHXD relieved TBI‐induced inflammation caused by damage to the chemical barrier of the intestinal mucosal barrier, which was nullified in the absence of CX3CR1 and CD36

Disruption of the biological barrier of the IMB worsens gut inflammation, which injures the integrity and function of the IMB's chemical barrier. In the healthy state, goblet cells can secrete sufficient mucus, and the mucus layer has abundant antimicrobial peptides that can efficaciously sustain the balance between ECs and gut bacteria and impede the occurrence of infection.^41^ The pathological characteristics of TBI‐induced IMB chemical barrier disruption are not only a reduction in goblet cells but also the destruction of the mucus layer and a reduction in mucus and ECs by bacteria, which magnifies gut inflammation.^41^ Mice indeed displayed chemical barrier destruction after TBI induction, and our study proved that TQHXD intervention could significantly restrain TBI damage to the chemical barrier in the mice. However, the therapeutic effect of TQHXD disappeared in the absence of CD36 and CX3CR1.

### 4.3 TQHXD maintained the integrity of the intestinal mucosal barrier's epithelial barrier by regulating innate and acquired immunity, but not when CX3CR1 and CD36 were deficient

The IMB immune barrier is composed of resident macrophages, lymphocytes, and DCs, which work together with the epithelial barrier to form the first line of intestinal defense. In this process, macrophage‐mediated innate immunity and Th_1_/Treg/Th_17_‐mediated adaptive immune response play vital roles in maintaining IMB function and the inflammation resolution response.^42^, ^43^ M1 macrophages are known to play pro‐inflammatory roles, while M2 macrophages play anti‐inflammatory roles.^44^ An ever‐increasing number of studies have found that promotion of gut epithelial repairment, elevated levels of ZO‐1 and occludin, and regulation of M1/M2 macrophage ratio and T‐cell subsets contribute to attenuation of gut inflammatory response.^30^, ^42^ Our results showed that TQHXD administration rectified the expression of epithelial proteins of ZO‐1 and occludin, M1/M2 macrophage ratio, protein and mRNA levels of iNOS and ARG1, and Th_17_/Treg and Th_1_/Treg balance in mice with TBI‐induced intestinal inflammation and IMB damage. These data implied that TQHXD could promote restoration of the IMB's epithelial barrier and regulate colonic innate and adaptive immunological reaction to exert protective effects.

CX3CR1^+^ macrophages are essential for maintaining gut immune homeostasis in response to systemic inflammation. It has been confirmed that the occurrence of gut inflammation, as the first line of defense in the intestinal immune response, can affect CX3CR1^+^ macrophage count, which can indicate the severity of inflammation in mice with colitis.^45^, ^46^ Ayush et al.^47^ demonstrated that experimental colitis could elevate CX3CR1^+^ macrophagic colonization in colon tissue. As a scavenger receptor, CD36 is essential for maintaining gut homeostasis, and its abnormal expression causes a defective IMB epithelial barrier^48^ and affects the resolution of inflammation by regulating the expression of CX3CR1^+^ macrophages.^49^ Based on the above‐described functions of CD36 and CX3CR1^+^ macrophages, we speculated that CD36 could be involved in the process of gut inflammation via modulation of CX3CR1^+^ macrophage expression. Therefore, we measured the expression of CD36 using WB and RT‐PCR and found that its levels in colon tissue were elevated in the TBI group and further increased in the TQHXD group compared with the TBI group. This indicated that CD36 exerted a protective effect in the pathological process of TBI‐induced GI dysfunction. The findings of Cifarelli et al.^48^ were similar to this result. Next, we used SSO, an inhibitor of CD36, on macrophage surfaces to treat mice with TBI‐induced GI dysfunction and discovered via a reduction in CX3CR1^+^ macrophage count that the therapeutic effect of TQHXD disappeared. This aggravated the pathological score; upregulated pro‐inflammatory factors such as IL‐1β, IL‐6, and TNF‐α; downregulated the anti‐inflammatory factor IL‐10; disrupted Treg/Th_1_ and Treg/Th_17_ balance; increased the M1/M2 macrophage ratio; and inhibited positive expression in ZO‐1, occludin, and PAS staining. In addition, we used CX3CR1^GFP/GFP^ and CX3CR1^GFP/+^ mice to clarify whether TQHXD exerted its protective effects through the regulation of CX3CR1. Surprisingly, we found that TQHXD indeed lost its regulatory role in the absence of CX3CR1, strengthening our finding that TQHXD could exert therapeutic action by activating CD36 and CX3CR1 on macrophage surfaces in mice with TBI‐induced intestinal inflammation and IMB damage, thereby elevating CX3CR1^+^ macrophage count; lowering the pathological score; downregulating pro‐inflammatory factors IL‐1β, IL‐6, and TNF‐α; upregulating anti‐inflammatory factor IL‐10; reducing Treg/Th_1_ and Treg/Th_17_ balance; decreasing the M1/M2 macrophage ratio; and promoting positive expression in ZO‐1, occludin, and PAS staining. Both CD36 and CX3CR1 insufficiency worsened GI dysfunction induced by TBI, which could not be rescued by TQHXD. However, not all study results are consistent with this: Lavalette et al.^49^ showed that mice lacking both CD36 and CX3CR1 on macrophage surfaces had a more severe inflammatory response. This might have been due to the different models and stages between the two studies.

### 4.4 TQHXD maintained the integrity of the intestinal mucosal barrier's biological, chemical, epithelial, and immune barriers by stimulating CD36/NR4A1/15‐LO, which was nullified when CX3CR1 was deficient

The transcription factor NR4A1 has been identified as a regulator that locates perivascular macrophages and can affect the function of CX3CR1^+^ macrophages by rectifying dysbacteriosis in the intestinal mucosa.^50^ To respond to inflammation, macrophages secrete 15‐LO, which is both pro‐inflammatory and anti‐inflammatory in leukocytes.^51^ Yang et al.^52^ showed that CX3CR1^+^ macrophages strongly upregulated 15‐LO in a hepatitis model, a trend that was reversed in CX3CR1–diphtheria toxin receptor (DTR) mice. Conversely, Merched et al.^53^ confirmed that 15‐LO overexpression in macrophages resulted in the production of anti‐inflammatory mediators during the degradation phase of inflammation. Based on our previous study showing that CX3CR1 deficiency aggravated the white‐matter injury and affected CD36/15‐LO/NR4A1 signaling in brain tissue of TBI mice,^17^ we speculated that CX3CR1 could also affect CD36/NR4A1/15‐LO signaling in gut tissue of TBI mice. In the present study, NR4A1 and 15‐LO proteins and mRNA levels were remarkably elevated in TBI group mice and reduced in TQHXD group mice. However, the expression of NR4A1 and 15‐LO in the SSO group was further elevated compared with the TBI group, while there was no significant difference between the SSO and TQHXD groups. In addition, we saw no significant difference between the CX3CR1^GFP/+^ + Sham and CX3CR1^GFP/GFP^ + Sham groups; compared with the CX3CR1^GFP/GFP^ + TBI group, levels of NR4A1 and 15‐LO showed no obvious change in the CX3CR1^GFP/GFP^ + TQHXD group, while such levels were further increased in the CX3CR1^GFP/GFP^ + SSO group. These results indicated that TQHXD could exert therapeutic action by reducing NR4A1 and 15‐LO levels in mice with TBI‐induced intestinal inflammation and IMB damage and that both CD36 and CX3CR1 insufficiency worsened GI dysfunction induced by TBI, which could not be rescued by TQHXD.

Taking these results together with our previous findings that TQHXD exerted therapeutic action by activating CD36 and CX3CR1 on macrophage surfaces in mice with TBI‐induced intestinal inflammation and IMB mucosal barrier damage, we inferred that TQHXD's mechanism of action was stimulation of CD36/NR4A1/15‐LO signaling in mice with TBI‐induced intestinal inflammation and IMB mucosal barrier damage by regulating the IMB's mucosal biological, chemical, epithelial, and immune barriers. This effect was nullified when CX3CR1 was deficient.

## 5 CONCLUSION

In this study, our data demonstrated that TQHXD exerted therapeutic effects on TBI‐induced intestinal inflammation and IMB damage by regulating the IMB's biological, chemical, epithelial, and immune barriers. The relevant mechanism was the stimulation of CD36/NR4A1/15‐LO signaling, which was nullified when CX3CR1 and CD36 were deficient. Meanwhile, our results further verified that TQHXD might be a potential candidate drug in therapy for TBI‐induced GI dysfunction.

## AUTHOR CONTRIBUTIONS

Wenzhu Wang, Heng Fan and Chunzhu Wei designed the study. Chunzhu Wei, Feng Zhu and Jintao Yu performed the experiments. Chunzhu Wei, Feng Zhu and Jintao Yu analyzed the data. Chunzhu Wei, Feng Zhu, Fei Gao, Yuyi Yuan, Yanlong Zhang and Xinjie Liu wrote the manuscript. Wenzhu Wang, Heng Fan, Chunzhu Wei, Feng Zhu, Jintao Yu, Si Chu, Dandan Cui reviewed/edited the manuscript. All authors approved the final version of the manuscript.

## FUNDING INFORMATION

This study was supported by the National Natural Science Foundation of China (NO. 81904023).

## CONFLICT OF INTEREST STATEMENT

The authors declare no competing interests.

## Supporting information


Supinfo

## Data Availability

The data that support the findings of this study are available from the corresponding author upon reasonable request.
