# Supplementary material for: Tongqiao Huoxue Decoction ameliorates traumatic brain injury‐induced gastrointestinal dysfunction by regulating CD36/15‐LO/NR4A1 signaling, which fails when CD36 and CX3CR1 are deficient
Source: CNS Neurosci Ther. 2023 May 8;29(Suppl 1):161–84. doi: 10.1111/cns.14247 (PMC10314107; doi:10.1111/cns.14247)
Supplement: Supplementary file 1 — Supinfo [file CNS-29-161-s001.pdf]

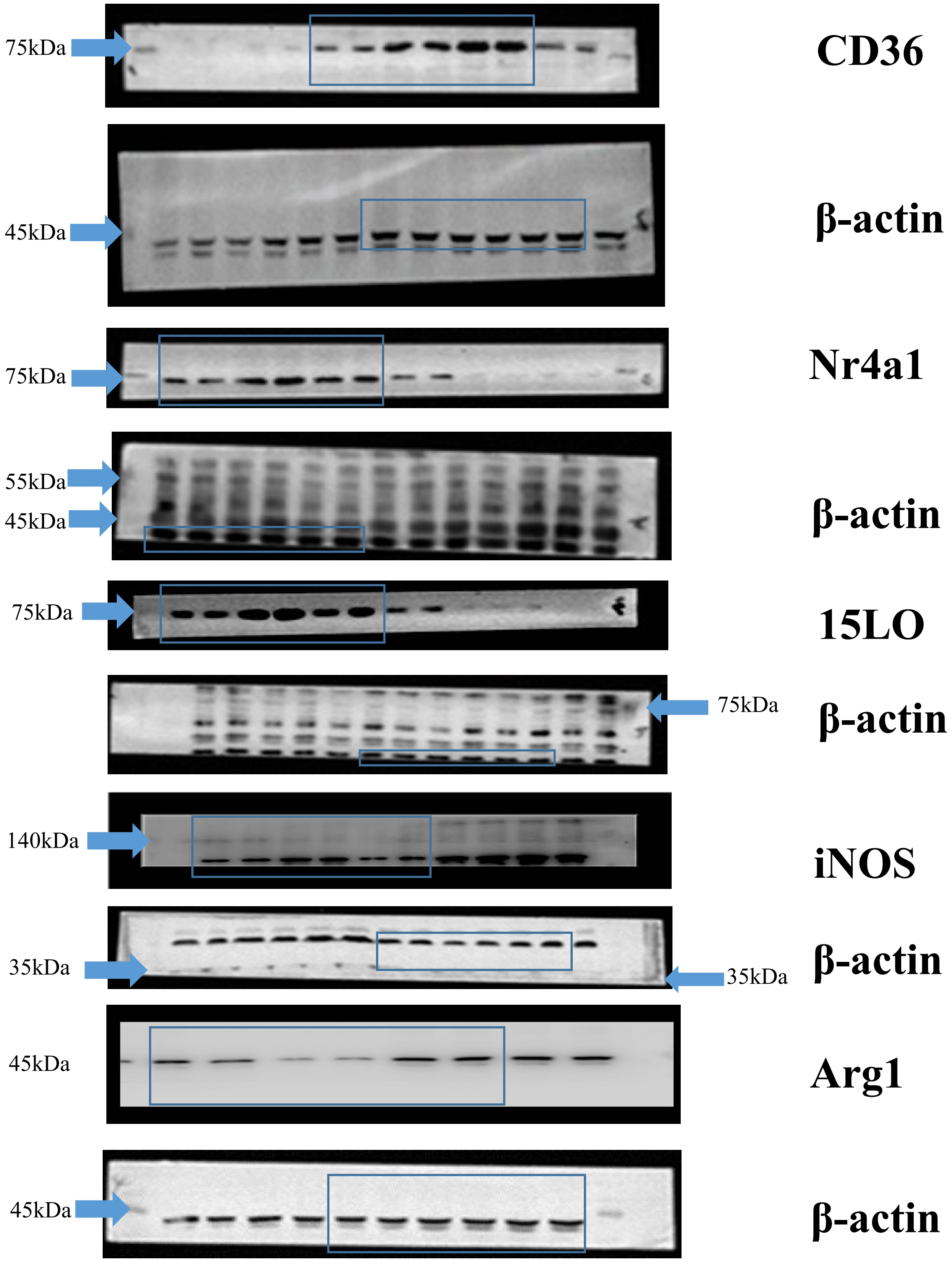

Full unedited gel/blot for Figure 6A-E in the manuscript

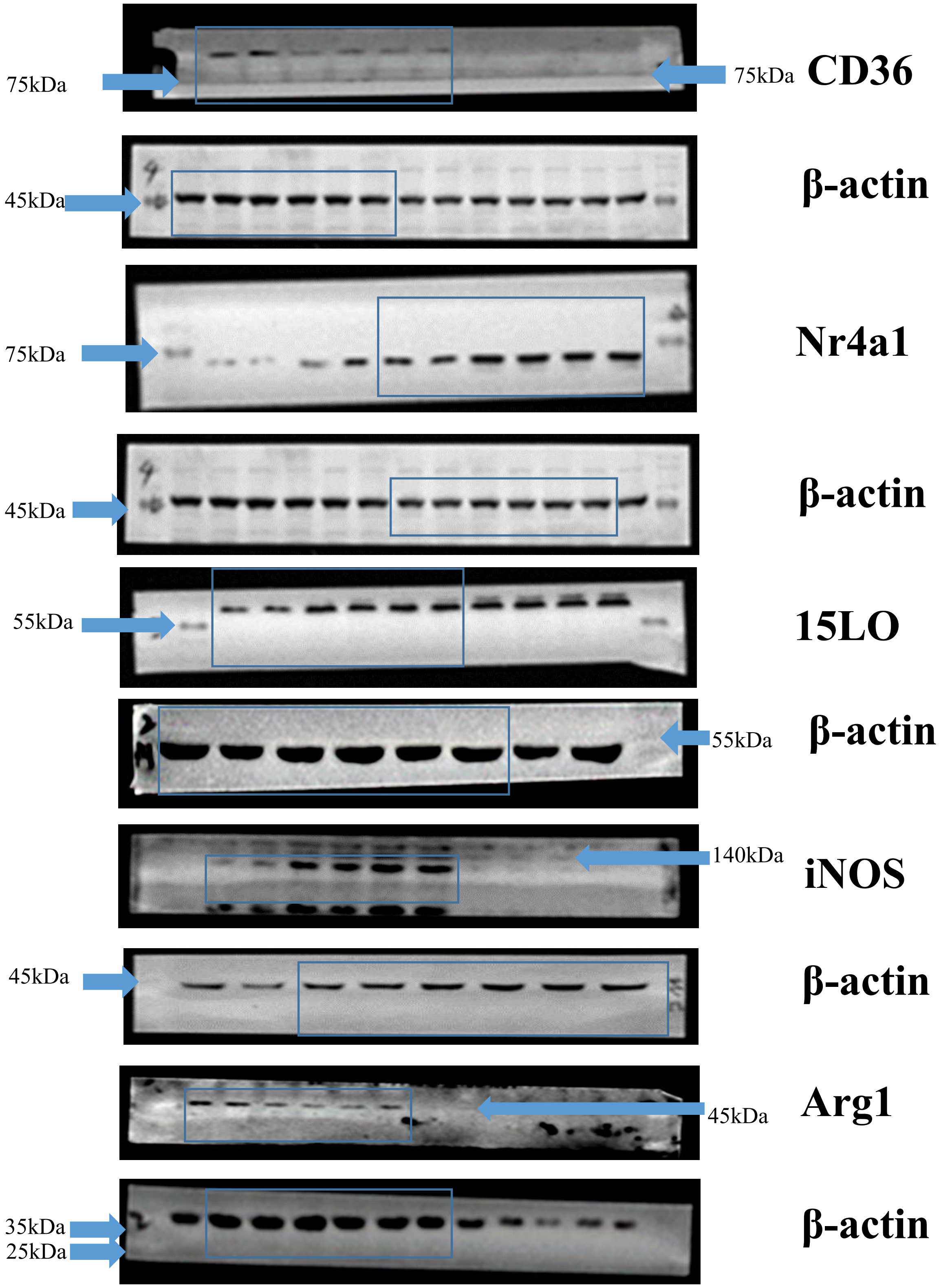

Full unedited gel/blot for Figure 8A-E in the manuscript

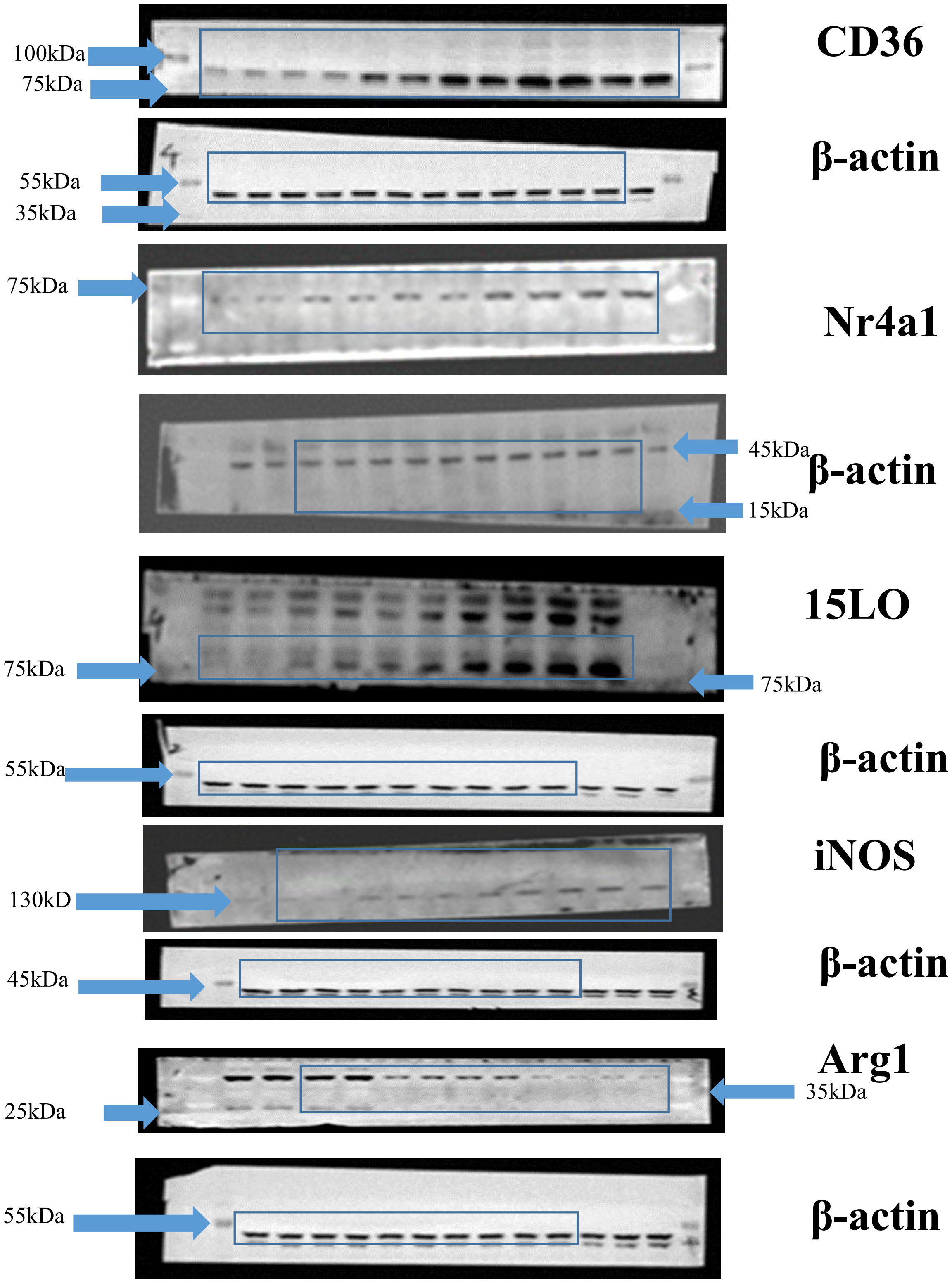

Full unedited gel/blot for Figure 14A，D, G, J, M in the manuscript
